# Recognition of a Mating Partner Using Cuticular Hydrocarbons in a Species with an Extreme Intra-sexual Dimorphism

**DOI:** 10.1007/s10886-025-01624-z

**Published:** 2025-07-17

**Authors:** Victoria C. Moris, Aline Wirtgen, Oliver Niehuis, Thomas Schmitt

**Affiliations:** 1 Department of Evolutionary Biology and Ecology, Institute of Biology I (Zoology), University of Freiburg, 79104 Freiburg, Germany; 2 Department of Animal Ecology and Tropical Biology, Biocentre, University of Würzburg, Am Hubland, 97074 Würzburg, Germany; 3 Marine Biology Lab, Université Libre de Bruxelles, 50 Av. FD Roosevelt, Brussels, 1050 Belgium

**Keywords:** Sexual attractiveness, Cuticular hydrocarbons, Methyl-branched alkanes, Anti-aphrodisiacs

## Abstract

Attracting a mate at the right time is critical for many species that reproduce sexually. In insects, short-range communication between potential mates is often mediated by cuticular hydrocarbons (CHCs), which cover most of the insect cuticle. Although the CHC profiles of many insects have been studied, we know little about what aspects of a CHC profile cause changes in mate attractiveness over the lifetime of an individual. We addressed this question by studying the mason wasp *Odynerus spinipes*, whose females exhibit age-related quantitative changes in their CHC profile composition. First, we created an ethogram of the male mating behavior. We observed in preliminary investigations that males do not attempt to mate with recently eclosed adult females. By coating wasp dummies with different CHC extracts, we were able to show that the CHC profiles of 0-day-old females are indeed less attractive to males than those of 3-day-old females. CHC profiles of 3-day-old females are characterized by significant decrease of the relative abundance of methyl-branched alkanes. These results, along with the consistently high relative abundance of methyl-branched alkanes in the CHC profile of males throughout their adult lifetime, suggest that in *O. spinipes*, methyl-branched alkanes may function as anti-aphrodisiacs. They likely reduce the harassment of females before they are ready to mate and help to prevent homosexual courtship.

**Supplementary Information:**

The online version contains supplementary material available at 10.1007/s10886-025-01624-z.

## Introduction

Chemical signaling has often been shown to be critical for successful reproduction in various animal species (Wyatt 2014; Blomquist and Ginzel 2021). In particular, insects are known to rely heavily on chemical communication for mate finding and recognition (Wyatt 2014). However, the mechanisms by which specific information, such as sexual attractiveness, is encoded in chemical cues have been poorly studied outside of *Drosophila* (Ayasse 2001; Allison and Cardé 2016; Stökl and Steiger 2017; Sun et al. 2023).

Cuticular hydrocarbons (CHCs), which cover most of the insect cuticle and protect against desiccation, play an important role in insect sexual communication (Dietemann et al. 2003; Steiger et al. 2011). In most insect species, each sex is characterized by a specific set of hydrocarbons of a certain length (typically 19 to 52 carbon atoms), which may contain double bonds and methyl branches. The pattern of abundance of different hydrocarbons on the cuticle is called the CHC profile. CHCs are known to be used by insects as contact pheromones (Ayasse et al. 2001; Paxton 2005; Ferveur 2005; Kroiss et al. 2006; Niehuis et al. 2013; Wyatt 2014). Qualitative and/or quantitative differences between the CHC profiles of different species and between the sexes of a given species allow individuals to identify conspecific mates (Thomas and Simmons 2008). However, the presence and abundance of specific components in a CHC profile can change over the lifetime of an individual (e.g., (Simmons et al. 2003; Hugo et al. 2006; Ichinose and Lenoir 2009; Nunes et al. 2009; Kuo et al. 2012; Polidori et al. 2017; Bien et al. 2019). These changes can be indicative of mating status and other traits and qualities of an individual (Paulmier et al. 1999; Ayasse et al. 1999; Schiestl and Ayasse 2000; Simmons et al. 2003; Mant et al. 2005; Grillet et al. 2006; Steiger et al. 2007; Everarets et al. 2010; Jennings et al. 2014). This, in turn, can influence an individual’s attractiveness (Kuo et al. 2012) and determine whether its CHC profile triggers courtship behavior in a potential mate (Finck et al. 2016; Würf et al. 2020). Although research on CHC-mediated mating behavior is limited, studies—mainly in the dipteran model *D. melanogaster*—have identified specific CHCs that are essential for mating, particularly single unsaturated compounds such as 7,11-dienes (Antony and Jallon 1982; Antony et al. 1985; Marcillac and Ferveur 2004; Grillet et al. 2006; Chertemps et al. 2006). Additionally, CHC profiles can change with age, potentially reducing the sexual attractiveness of mating partners (Kuo et al. 2012).

The mason wasp *Odynerus spinipes* (Eumininae: Vespidae) has proven to be a promising species for elucidating the molecular basis and evolution of CHC profile differences (Moris et al. 2023). Females of this species are known to be capable of expressing one of two CHC profiles, known as chemotypes, which they maintain throughout their adult lives (Wurdack et al. 2015; Moris et al. 2021). Both chemotypes are commonly found within the same population (Wurdack et al. 2015; Moris et al. 2021). The two chemotypes differ qualitatively in 77 CHCs, mainly in alkenes having their double bonds at uneven positions for chemotype 1 and at even positions for chemotype 2 (Wurdack et al. 2015; Moris et al. 2021). In contrast, *O. spinipes* males express only one CHC profile that is very similar to one of the two chemotypes expressed by their females (Wurdack et al. 2015). The composition of the two chemotypes expressed by *O. spinipes* females changes with female age (Moris et al. 2023), but the compositional changes and their functional role have not yet been investigated in detail. Males face the problem of recognizing females with both chemotypes as appropriate mating partner. This could be caused by two sets of compounds, both of which are attractive to males, or by a sex pheromone encoded in the CHC profile of both chemotypes. We hypothesize that females will show a sex pheromone during mating period in both chemotypes that is different from the male CHC profile.

In this study, we report our results from investigating age-related changes in the CHC profiles of O. *spinipes* females and assessing the extent to which these changes alter female attractiveness to conspecific males. Specifically, we (1) recorded the mating behavior of males and females to create an ethogram of the male courtship behavior. Since we know from a preliminary investigation that males do not attempt to mate with recently eclosed adult females, we (2) conducted male O. *spinipes* mating experiments using female O. *spinipes* dummies coated with CHC profiles of females of different ages. Specifically, we compared the attractiveness of CHC profiles of recently eclosed females (0 days old) with those of females that were 3 days old. Finally, given the different response of males to dummies coated with CHC profiles of females of different ages, we analyzed (3) in what aspects the CHC profiles of *O. spinipes* females (both chemotypes) and males (for the sake of completeness) change during the adult life of the wasps, sampling wasps 0, 3, 9 and 14 days after their eclosion.

## Results

### Male Mating Behavior

**Fig. 1:**
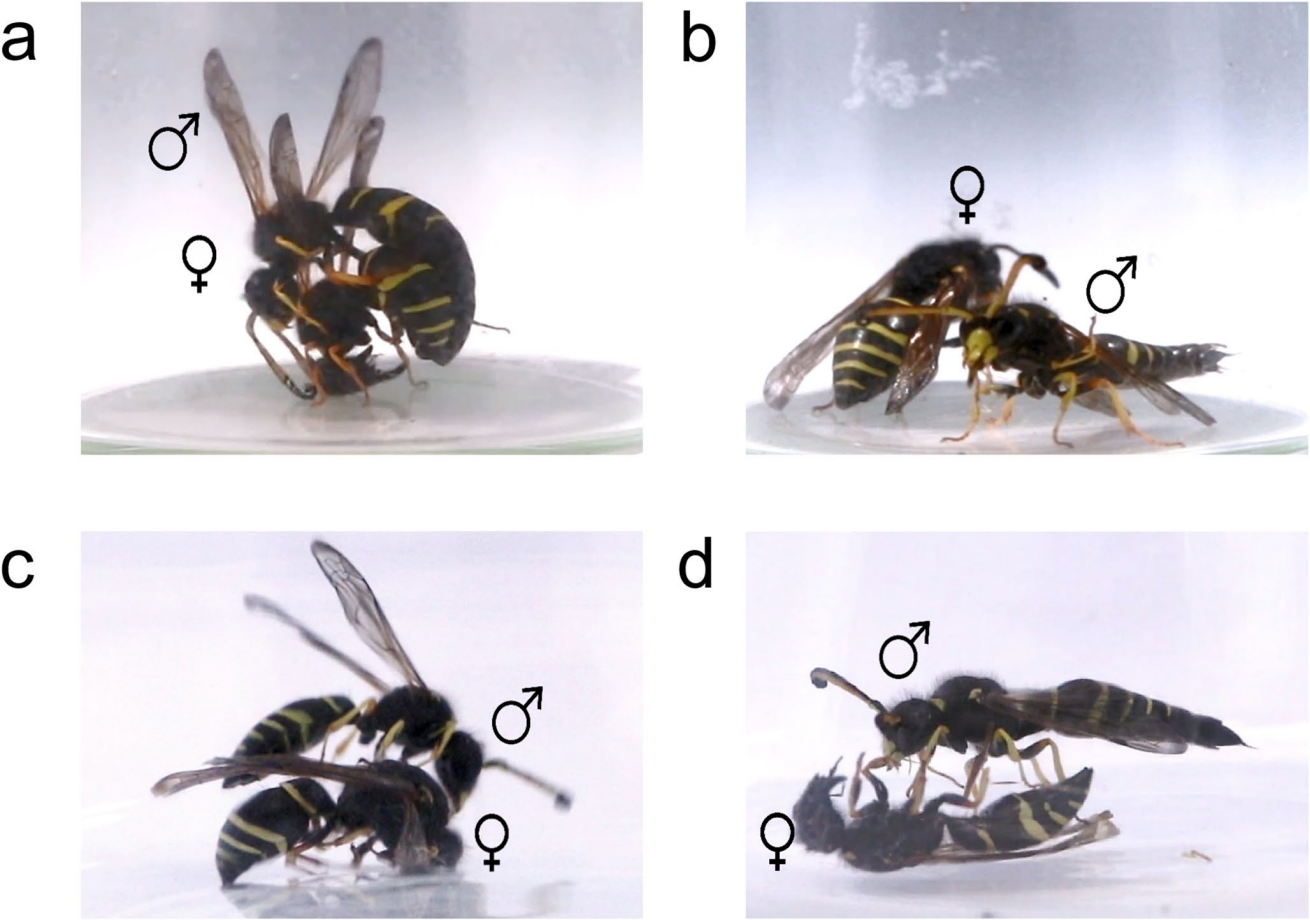
Comparison of *O. spinipes* male mating behavior when paired with living *O. spinipes* female (**a**, **b**) versus a dead dummy covered by CHC extracts (**c**, **d**): (**a**) *O. spinipes* male mounts a living female and loops the tips of its antennae around those of the female; (**b**) *O. spinipes* male extruding its genital capsule after having copulated with the female; (**c**) *O. spinipes* male mounts a dummy female; (**d**) *O. spinipes* male extruding its genital capsule after having copulated with a dummy female

We recorded the behavior of nine *O. spinipes* male-female pairs that had eclosed 4–5 days earlier (Fig. 1a; Table S1). We found that the male mating behavior (= B) is highly stereotyped and does not differ when males are presented with the two female chemotypes. This behavior can be summarized as follows (Fig. 1b–e): when the male physically meets the female, it (B1) makes antennal contact with the female. It then (B2) mounts the female and wraps its antennae around the female’s antennae. Once the female is mounted, the male (B3) extends its aedeagus and rubs it against the female’s metasoma, causing the female to open its genital opening. The male (B4) inserts its genital capsule into the female’s genital opening. After mating with the female, and even after unsuccessful mating attempts, the male (B5) lifts its metasoma and extrudes their genital capsule. All tested males exhibited behaviors B1, B2, and B3. In two cases, we did not observe behavior B4, and in one case, behavior B5, suggesting that mating may not have been entirely complete for these two males (Table S1).

### Effect of Female CHCs on Male Attraction

**Fig. 2:**
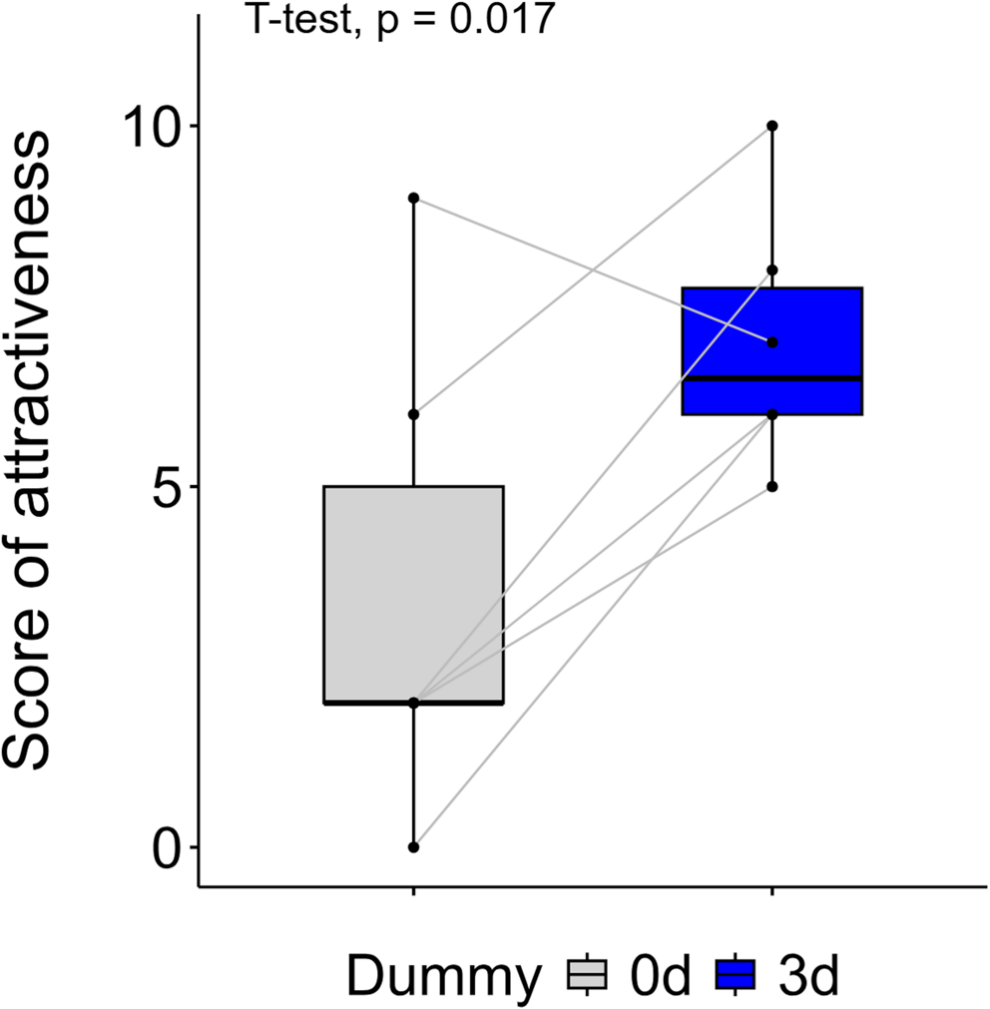
Attractiveness scores of female dummies coated with cuticular hydrocarbons extracted from 0-day- (gray box plot) and 3-day-old (blue box plot) females to six males. Gray lines indicate each pair of data. See Materials and Methods for how attractiveness scores were calculated

Based on preliminary observations (Fig. S1) showing that males do not mate with newly eclosed females, we conducted experiments using female dummies with manipulated CHC profiles. Specifically, we tested whether males are more attracted to dummies coated with CHC extracts from 3-day-old *O. spinipes* females compared to those from 0-day-old females. In trials where six males were sequentially presented (in randomized order) with two dummies representing different age-class CHC profiles but the same chemotype, we observed a significant preference (Fig. 2; one-sided paired t-test: *p* = 0.017; Cohen’s d = 1.19; post hoc power = 0.8; Table S2).

### CHC Profile Differences Between 0- and 3-day-old Females

**Fig. 3:**
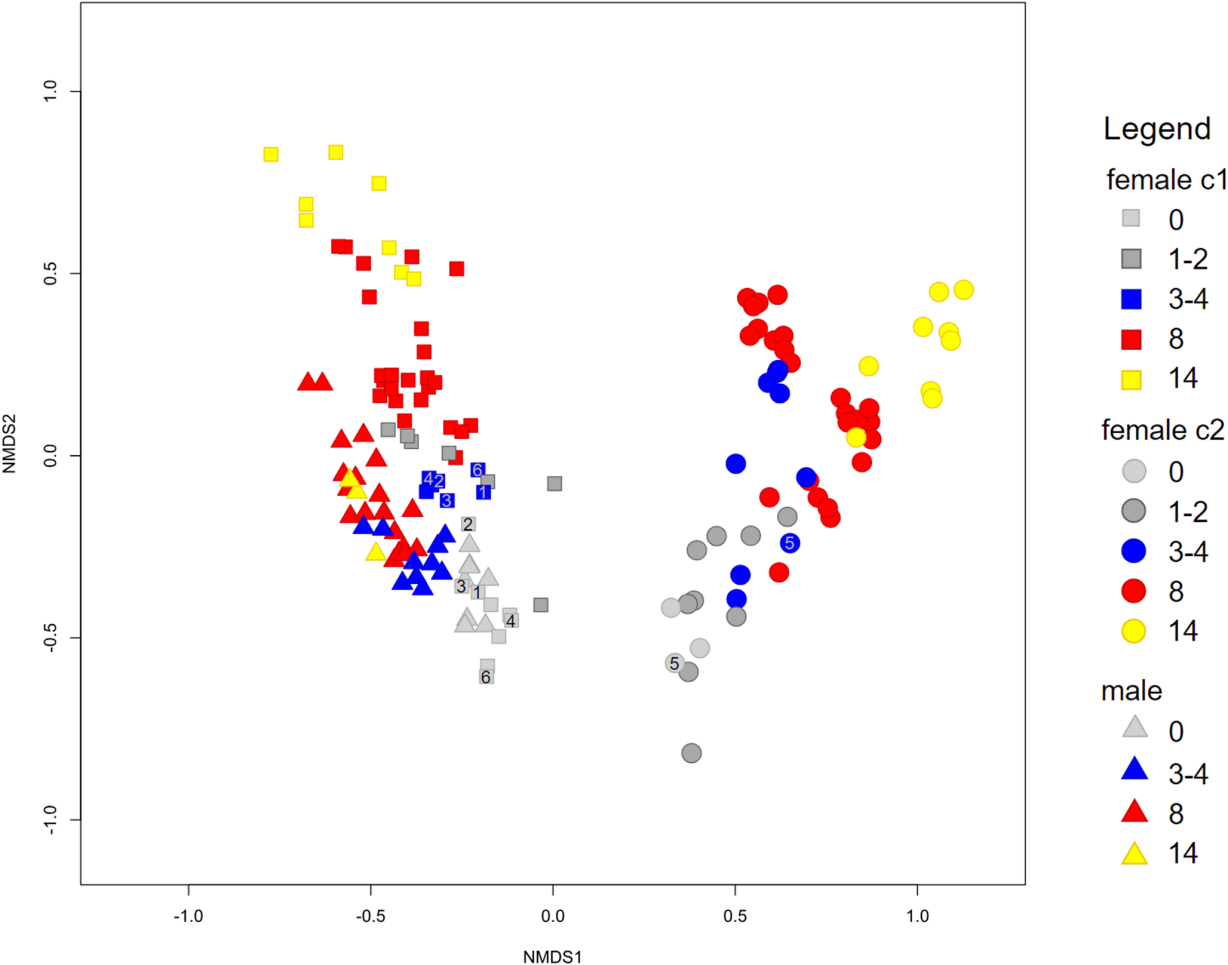
Non-metric multidimensional scaling (NMDS) ordination of Bray-Curtis distances between cuticular hydrocarbon (CHC) profiles of *O. spinipes* males and females. Each data point represents a CHC profile of an *O. spinipes* sample at a different time point: day 0 (gray), day 1–2 (dark gray), day 3–4 (blue), day 7–8 (red), and day 14–15 (yellow). CHC profiles of males (*N* = 40: N_0d_ = 8, N_3d_ = 10 N_8d_ = 19, N_14d_ = 3) are indicated by triangles, those of females of chemotype 1 (= c1; *N* = 55: N_0d_ = 9, N_1d_ = 7, N_3d_ = 7, N_8d_ = 24, N_14d_ = 8) by squares and those of chemotype 2 (= c1) females (*N* = 54: N_0d_ = 3, N_1d_ = 9, N_3d_ = 9, N_8d_ = 24, N_14d_ = 9) by circles. Samples used to cover dummy females are indicated by the ID of the male that had to choose between them (see Table S2)

We compared the composition of CHC profiles from *O. spinipes* wasps (Tables S3, S4) of different age classes (i.e., 0, 1–2, 3–4, 8, 14 days after eclosion), considering both female chemotypes and also the male sex. An NMDS plot of the Bray-Curtis distances of the CHC profiles (Fig. 3) showed that the samples of the two female chemotypes form two clearly distinct groups, with the CHC profiles of the males nested within the group of CHC profiles of the females with chemotype 1. Within each of the two groups, we found that the CHC profiles of female samples from a given age class are clustered and that samples from different age classes are located in different regions of the chemospace. Interestingly, the chemospace of young males largely overlaps with the chemospace of young females with chemotype 1.

**Fig. 4:**
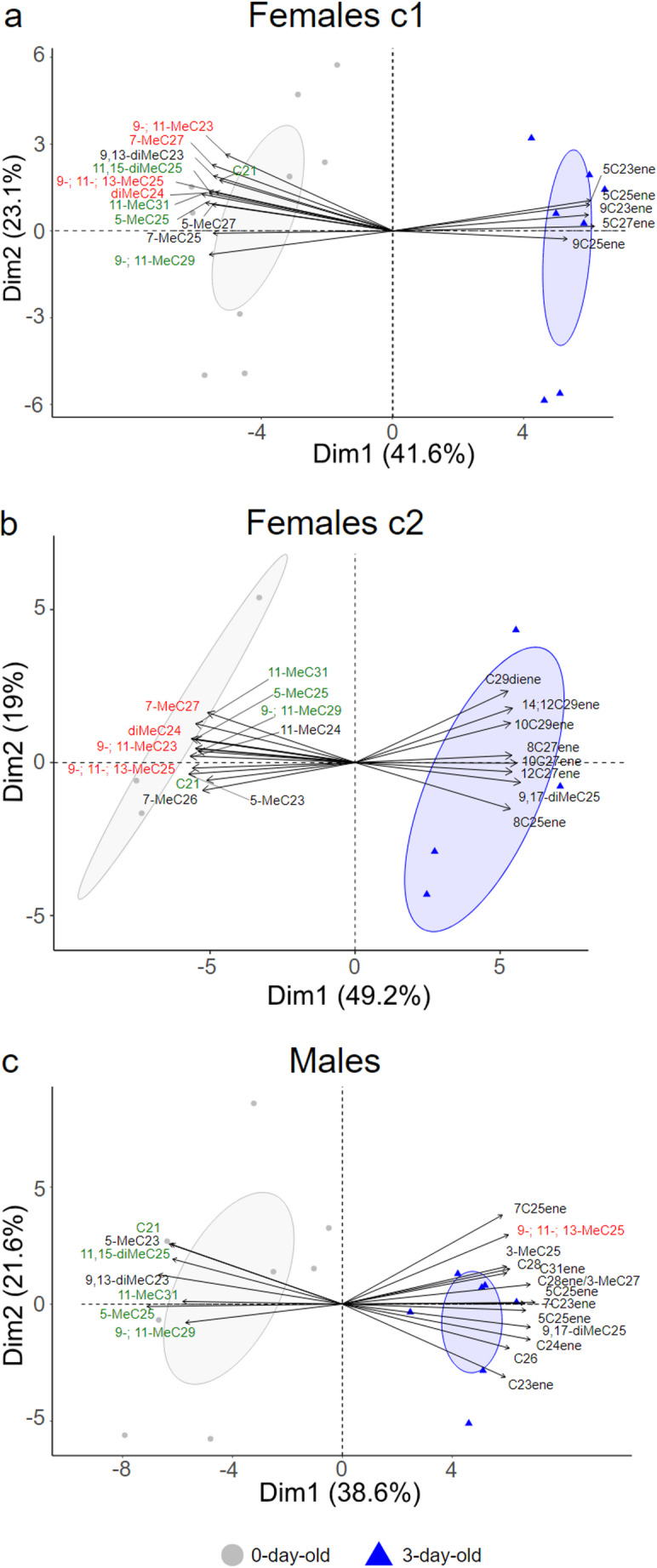
Principal component analysis (PCA) of cuticular hydrocarbon (CHC) profiles of *O. spinipes* females (chemotype 1 [= c1]: left; chemotype 2 [= c2]: middle) and of males, with the eigenvectors of those CHCs whose relative abundances are most correlated along the first principal component. Each marker represents a CHC profile of a wasp of a different age class (grey: day of eclosion; blue: 3 days after eclosion). CHCs that explain the differences between 0-day- and 3-day-old CHC profiles found in common in the two chemotypes but not in male CHC profile are written in red; CHCs that explain the differences between 0-day- and 3-day-old CHC profiles found in common in the two chemotypes and in male CHC profile are written in green

We used PCA to shed light on the identity of the CHCs driving the differences in CHC profiles between females of the two age classes of particular interest to us (i.e., 0 and 3 days after eclosion) of each chemotype. The first principal component explained a substantial amount of the total variation in CHC profiles (chemotype 1: 42%, chemotype 2: 49%) and clearly separated the CHC profiles of 0-day-old wasps from those of 3-day-old wasps in both analyses (Fig. 4). While the extraction method (SPME fiber, hexane) did not appear to strongly affect clustering in the NMDS plot, PCA analyses were performed exclusively on CHC samples extracted with hexane from the same year. This approach enhanced the power of the PCA analysis by minimizing potential variation due to different GC/MS systems or extraction methods (SPME vs. hexane) and enabled us to identify key compounds associated with age-related differences in CHC profiles. Analyzing the contributions of each compound to the variability in dimension 1, we found that certain compounds primarily explain the differences in CHC profiles between 0-day- and 3-day-old females, common across both chemotypes but absent in males. These compounds are methyl-branched alkanes: 11-; 9-MeC23, x, y-diMeC24, 13-; 11-; 9-MeC25, and 7-MeC27 (highlighted in red in Fig. 4). Other methyl-branched alkanes, common across both chemotypes and males, include 11,15-diMeC25, 5-MeC25, 11-; 9-MeC29, and 11-MeC31 (in green in Fig. 4). Additionally, alkenes contribute to the differences between 0-day- and 3-day-old female CHC profiles, though they vary between chemotypes by double bond position—uneven carbon numbers in chemotype 1 and even carbon numbers in chemotype 2. Some of these alkenes are also present in males (Fig. 4).

**Fig. 5:**
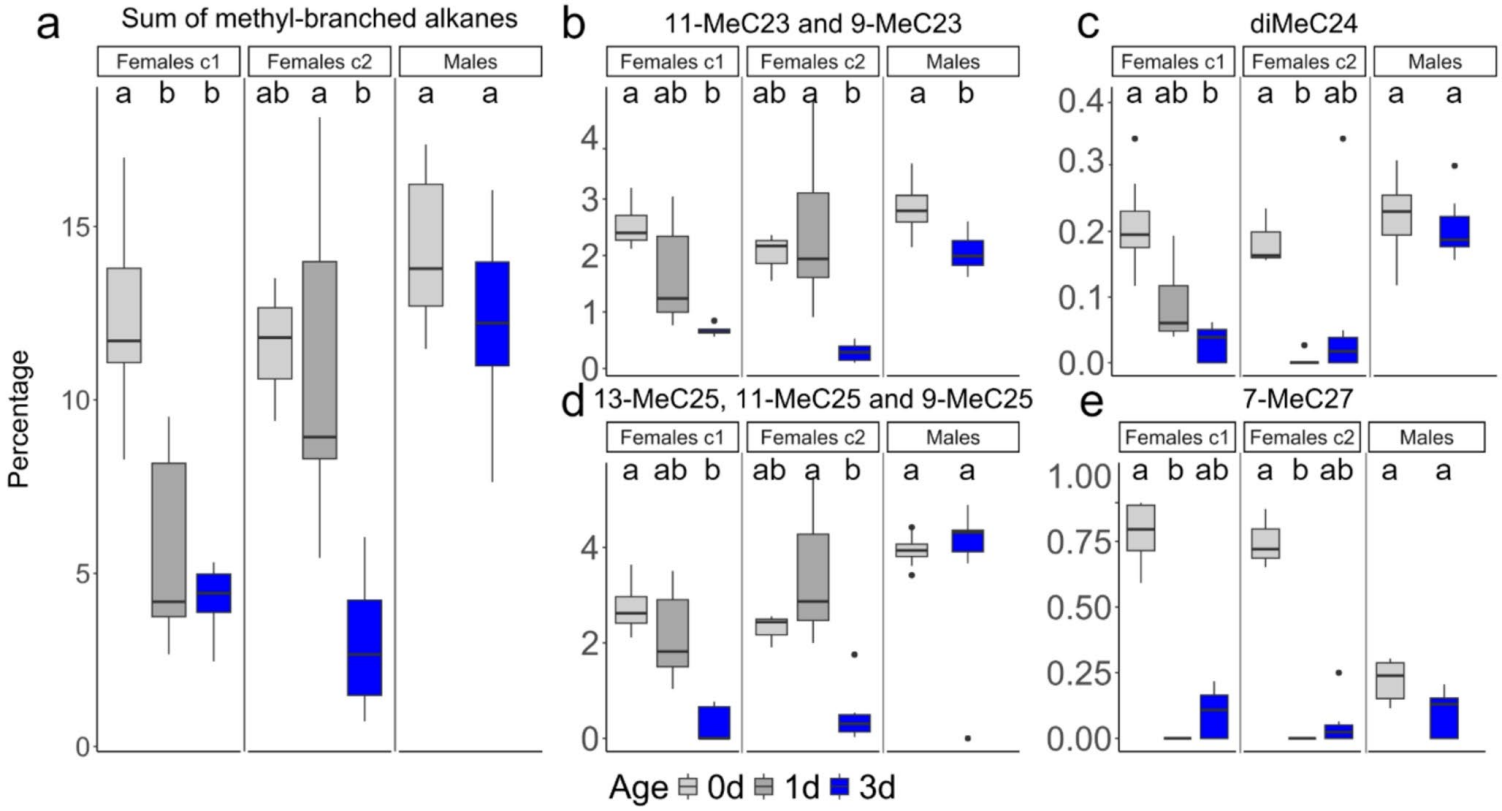
Relative abundance of methyl-branched alkanes in *O. spinipes* males and females (c1 = chemotype 1; c2 = chemotype 2) on the day of eclosion (gray) and 3 days after eclosion (blue). The ordinate shows the relative abundance (in percent) of all methyl-branched alkanes (**a**), of 11-MeC23 and 9-MeC23 (**b**), of diMeC24 (**c**), of 13-MeC23, 11-MeC23 and 9-MeC23 (**d**), 7-MeC27 (**e**). Letters indicate the significant differences between groups (*p*-value < 0.05). Individual *p*-values and adjusted *p*-values are provided in Table S5

Given the PCA results, we examined at the overall differences in the abundance of methyl-branched alkanes in the CHC profiles of 0-day- and 3-day-old wasps. Among the two age classes, we found that methyl-branched alkanes differed the most: more than 70% decrease in abundance in the profiles from day 0 to day 3 (chemotype 1: day 0, mean 12.3%, *N* = 9, day 3, mean 3.7%, *N* = 7; chemotype 2: day 0, mean 11.6%, *N* = 3, day 3, mean 3.0%, *N* = 9). For comparison, males maintained a relatively high abundance of methyl-branched alkanes in their CHC profile over the same time period (day 0, 14.3%, *N* = 8, day 3, 12.3%, *N* = 10; Table S4). Indeed, the total abundance of methyl-branched alkanes was significantly reduced for females of chemotype 1 (Dunn post-hoc test, Bonferroni adjusted *p* = 0.0085) and for females of chemotype 2 (Dunn post-hoc test, Bonferroni adjusted *p* = 0.019 between 1-day-old and 3-day-old females) 3 days after eclosion, while it was not significantly different in 0-day- and 3-day old males (Fig. 5, Table S5). The test was significant in females of chemotype 2 when comparing 3-day-old females with 1-day-old with, and not with when comparing 3-day-old females with 0-day-old females, possibly due to a limited sample size of the latter group (*N* = 3; Fig. 3).

Examining the relative abundance of methyl-branched alkanes identified in PCA analyses of females of chemotypes 1 and 2, but absent in males, we observed two distinct groups of compounds that significantly decreased in females of both chemotypes three days after eclosion (Fig. 5, Table S5): 11-; 9-MeC23, and 13-; 11-; 9-MeC25. The difference was significant in females of chemotype 2 when comparing 1-day-old with 3-day-old females, likely because of smaller sample size of 0-day-old females of chemotype 2, as explained above. In males, these compounds maintained a high relative abundance, although the first compound also showed a slight decrease—not as pronounced as in females. These methyl-branched alkanes constitute a major proportion of the overall profiles in both males and females. We also observed a significant decrease in x, y-diMeC24 levels in 3-day-old females of chemotype 1 compared to newly eclosed females and in 1-day-old females of chemotype 2 compared to newly eclosed females but not in 3-day-old females, even if the percentage was lower. The percentage of that compound did not change in males. Additionally, 7-MeC27 levels significantly declined already in 1-day-old females of chemotype 1 compared to their levels at eclosion (although the decrease is not significant both chemotypes 3 day after eclosion), with this compound being less abundant in males than in newly eclosed females. Among other highly abundant methyl-branched alkanes (Fig. S2), we found that 3-MeC25 and 3-MeC27 were unique in showing an increase in males, while the remaining compounds declined in both males and females.

## Discussion

We examined age-related changes in the CHC profiles of O. *spinipes* females and assessed the extent to which these changes alter female attractiveness to conspecific males. *O. spinipes* is a particularly interesting species in this regard, as its females can express one of two chemotypes that differ in 77 compounds (Wurdack et al. 2015). Since we observed mating with females of both chemotypes (Table S1, Fig. S1), it appears that males are attracted to females of both chemotypes. Moreover, by conducting dummy experiments with dead females, we could show that both chemotypes elicit courtship behavior in males suggesting that a short-range female sex pheromone is encoded in the CHC profile of mature females. Therefore, if females of the two chemotypes show a similar age-related change in attractiveness to conspecific males, commonalities in their CHC profile changes could point to the causal compound(s).

Using initially odorless dead wasps coated with CHCs extracted from females of different ages and chemotypes, we found that CHC extracts from 3-day-old females of both chemotypes were statistically significantly more attractive to males than those from recently eclosed females. Although the sample size was limited, reflecting the challenges of working solitary wasps, the detection of a statistically significant effect—with an estimated post hoc power of approximately 80%—suggests a robust and convincing effect size. The main difference in CHC composition between recently eclosed and 3-day-old females is the relative abundance of certain methyl-branched hydrocarbons and alkenes. Specifically, methyl-branched alkanes present in the CHC profiles of recently eclosed females are almost absent in females 3 days old or older. The profiles of these older females are also characterized by a relatively high abundance of unsaturated hydrocarbons. The low attractiveness of recently eclosed females may result from a low relative abundance of unsaturated hydrocarbons on their cuticle, a high relative abundance of methyl-branched hydrocarbons, or a combination of both. Although our data do not allow us to test these two hypotheses directly, several observations in this study support the second hypothesis, suggesting that methyl-branched hydrocarbons may act as anti-aphrodisiacs.

First, we found that males consistently exhibit a high relative abundance of methyl-branched hydrocarbons throughout their lives. In addition, over the course of a year-long study, we observed no instances of males attempting to mate with other males. Taken together, these findings suggest that methyl-branched hydrocarbons may serve as chemical cues that enable males of *O. spinipes* to distinguish potential mates from non-mates, supporting their role as anti-aphrodisiacs. These compounds are abundant in both males and newly-emerged females, rendering both unattractive to mating males. Second, methyl-branched alkanes are the only compounds that change in relative abundance in both females of chemotype 1 and chemotype 2. In contrast, alkenes differ between the two chemotypes, making the first hypothesis proposed in the introduction less likely, as males would require different receptors to perceive alkenes with double bonds at different carbon positions. Finally, the idea of methyl-branched hydrocarbons acting as anti-aphrodisiacs in very young females is more plausible, as we consistently recorded substantial quantities of unsaturated hydrocarbons in young females, making this compound less likely to trigger mating behavior in males.

Only specific methyl-branched alkanes seem likely to serve as anti-aphrodisiacs. The relative abundance of some methyl-branched alkanes decreases in both males and females (Fig. S2), making these compounds less likely candidates for this role. In contrast, 11-MeC23, 9-MeC23, 13-MeC25, 11-MeC25, and 9-MeC25 are promising candidates, as they represent the majority of the methyl-branched alkanes in both males and females. Notably, their relative abundance is high in males and in newly eclosed females, yet decreases in females, nearly disappearing three days after eclosion, while remaining high in males. Interestingly, these compounds are structurally similar, differing only by two carbons, suggesting they may be recognized by similar receptors and act as the primary methyl-branched alkanes with an anti-aphrodisiac function in *O. spinipes*.

Changes in the abundance of methyl-branched alkanes in CHC profiles have been reported in other species, one of which has also been linked to female attractiveness. Immature *D. melanogaster* and *Drosophila simulans* express more methyl-branched alkanes than mature flies (Pechine et al. 1988). Female *Chrysomya putoria* flies (Braga et al. 2016) also express more methyl-branched and more dimethyl-branched alkanes, than mature flies. In *Nasonia vitripennis*, the abundance of methyl-branched alkanes in the female CHC profile correlates with female attractiveness. However, in contrast to *O. spinipes*, this correlation is positive (Sun et al. 2023).

Methyl-branched alkanes, functioning as anti-aphrodisiac, would enable males to distinguish between males and females, thereby preventing homosexual courtship. Additionally, they would reduce male harassment of younger females. The high abundance of methyl-branched alkanes in very young *O. spinipes* females could represent a mimicry of the male CHC profile, reducing male harassment of females before they are ready to mate. Similar cases of CHC profile mimicry have been observed in Hymenoptera and rove beetles. In these cases, the immature males mimic female CHC profiles to avoid harassment from competing males (Peschke 1985, 1987a, b; Steiner et al. 2005). Avoidance of male harassment after mating has also been observed in the digger wasp species *Stizus continuus*, and is associated with changes in female CHC profiles, rather than the transfer of compounds by males during mating (Polidori et al. 2017). Mimicry of male CHC profiles by females has already been observed in *D. melanogaster*. In this species, compounds like Z7-tricosene, a major component of the male cuticle (Scott 1986), are transferred to females during mating. Future studies could explore whether dummies coated with male CHC extracts are less attractive to males, providing evidence that males are able to distinguish between sexes based solely on CHC profiles.

In addition to a potential anti-aphrodisiac in eclosed females, a sex pheromone from 3-day-old females likely initiates the courtship behavior of the males. The other candidates to act as sex pheromones are the alkenes which increase significantly with age in females and are absent or present at lower levels in males. However, not the same ones increase in the two chemotypes: shorter alkenes (23–25 carbons) with double bond at uneven positions in chemotype 1 and longer ones (27–29 carbons) with double bonds at even positions in chemotype 2 (Fig. 4; Fig. S3; Moris et al. 2021; 2023). This makes it unlikely that a specific alkene is the pheromone, as has been shown for alkadienes with specific double bond positions and chain lengths in *D. melanogaster* (Ferveur 2005).

In this study, we demonstrate that age-related changes in the CHC profiles of *O. spinipes* females trigger male courtship behavior. The reduction of methyl-branched hydrocarbons likely increases female attractiveness, suggesting that these compounds have an anti-aphrodisiac effect. We propose that, in *O. spinipes*, female attractiveness is driven by the reduction of a repellent class of compounds. This study highlights the role of methyl-branched alkanes in chemical signaling. While these compounds are crucial for chemical communication, particularly in Hymenoptera, their genetic basis remains largely unexplored (Sun et al. 2023). By highlighting the importance of these compounds in the solitary wasp *O. spinipes*, a species with a recently published genome, this study provides a strong foundation for future research into their genetic underpinnings.

## Materials and Methods

### Collection and Rearing of Odynerus Spinipes

We collected O. *spinipes* in 2016, 2018, and 2019 from trap nests installed in Büchelberg (Germany, Rhineland-Palatinate, WGS 84: 49.027985, 8.164801) (for more information, see (Moris et al. 2023). We opened the trap nests in March and April to collect *O. spinipes* prepupae. The prepupae were stored in hard gelatin capsules (size 3, LUTOR, Cologne, Germany, and Birmingham, UK) at 5 °C for 2–4 weeks. Prior to the experiments, the gelatin capsules containing the prepupae were placed in a dark climate chamber at 23 °C to induce eclosion. To avoid sample loss due to desiccation, the gelatin capsules containing the prepupae were always kept in a closed polystyrene box with humidified peat, which we changed every two days. After hatching, the wasps were transferred to observation cages with a viewing window and zipper (30 cm x 30 cm x 30 cm; Bioform, Nuremberg, Germany), which we kept in a climate chamber at 21 °C, 70% relative humidity, and a 14.5/9.5 h day/night cycle. All wasps were fed with honey water in a petri dish lined with white paper. A total of 144 *O. spinipes* wasps were reared by us over three years. Of these, 40 were females with chemotype 1, 36 were females with chemotype 2, and 68 were males (most of them had their CHC profiles analyzed: see Table S6). In addition to wasps sampled in Germany, we collected female wasps in in 2017 in Tenneville (Belgium; WGS 84: 50.100671, 5.530061) and stored them in the freezer (-20 °C) before further processing them so we could use them as dummies for behavioral experiments.

### Male Mating Behavior

To gain an understanding of the mating behavior of *O. spinipes* males, we recorded the behavior of nine *O. spinipes* males that had eclosed 4–5 days earlier, in the presence of age-controlled females. The females were kept separately in individual observation cages with food (paper with honey and water) in a climate chamber (70% humidity, 21 °C, 9.5 h dark and 14.5 h light) prior to the experiments. The males were housed under identical conditions, with the exception that they were kept in pairs due to space constraints. The cages appeared sufficiently large to minimize frequent interactions between the two males. Additionally, since males raised in isolation exhibited the same behavior toward female dummies, this suggests that their paired housing had no significant impact on their behavior. The experiments were performed with wasps that had been exposed to 6.5 h of light that day, which in mid-May in southern Germany corresponds to about 11:30 a.m., the time at which we observed samples of the species mating in the field. We started the experiments by placing a given female in a glass tube (inner diameter = 25 mm, height = 50 mm, volume = 25 ml) into which the male was introduced 5–10 s later. The tube was surrounded by white cardboards to create a uniformly illuminated environment for the wasps (Fig. 1a). A small hole in the cardboard on one side was used to record the wasp’s behavior using an EOS 5D Mark IV in video mode (Canon, Tokyo, Japan). Females and males were kept together and videotaped for 30 min. The behavior of the male wasp on the video recordings was then analyzed to derive an ethogram.

### Behavioral Bioassays

To assess whether differential attraction to females of different ages is mediated by the CHC profile of the females, we conducted behavioral assays in which we offered *O. spinipes* males initially odorless dead female wasps that we coated with CHC extracts from 0-day-old and 3-day-old females. The females that we used as dummies were collected in 2017 in Tenneville (Belgium; WGS 84: 50.100671, 5.530061) and stored in the freezer (-20 °C) before further processing. The dummies were treated with dichloromethane in a Soxhlet extractor for at least ten cycles to remove their CHCs and other potential chemical cues. The wasps were then placed under a fume hood for 24 h to ensure that the solvent had evaporated. To coat the dummies with the CHC profiles of females of a given age, we collected CHC extracts (see below for details) from 0-day-old and 3-day-old females reared in 2019 from the trap nests at the Büchelberg field site. An aliquot of each CHC extract was used for CHC profile characterization by GC-MS (see also Fig. 3). The remaining volume of CHC extract was reduced to 200 µl by evaporation of excess solvent under a gentle nitrogen stream. The 200 µl of CHC extract was carefully applied drop by drop to the dummies. The dummies were placed under a fume hood overnight to allow the solvent to evaporate. A total of 18 female dummies were prepared: seven were coated with CHC extract from 0-day-old chemotype 1 wasps, two with CHC extract from 0-day-old chemotype 2 wasps, seven with CHC extract from 3-day-old chemotype 1 wasps, and two with CHC extract from 3-day-old chemotype 2 wasps. The perfumed female dummies were presented to 4–5-day old male wasps reared in 2019 from the same trap nests as the females whose CHC extracts we used to perfume the female dummies. To facilitate the preparation of the dummies and the analysis of the CHC extracts used to coat them, male development was delayed by keeping them at 5 °C for an additional 1–2 weeks. Once they eclosed, unmated males used in the bioassays were kept individually in cages.

Behavioral assays were performed as follows: a female dummy was placed with forceps (cleaned with dichloromethane between each experiment and dried under a fume hood) in the center of the bottom of an Erlenmeyer flask (50 mL volume, 85 mm height, 29 mm opening diameter) and surrounded by white cardboards (Fig. 1A). After 6.5 h of light exposure on the day of the experiment, a male wasp was placed in the flask and its behavior was videotaped for 10 min., beginning with the first antenna contact of the male with the female dummy. After a break of 24 h, we repeated the experiment, this time offering the male a female dummy coated with a different CHC extract. Half of the males were first presented with a dummy coated with a CHC extract from a 0-day-old female, followed by a dummy coated in a CHC extract from a 3-day-old female. For the other half, the order was reversed. We confirmed that the attractiveness scores did not differ significantly between dummies presented first and those presented second (Fig. S4). Each dummy was used only once. In order to have only the age of the female influence any differential attraction by the male, a given male was only provided with CHC extracts from females of the same chemotype. In total, we analyzed the behavior of nine males when they were presented sequentially with two dummies. In six of the nine trials, the male exhibited mating behavior with at least one of the presented dummies. Only the behaviors from these trials were scored. Indeed, three males did not show any mating behavior with either with the first or the second dummy, making it impossible to compare their responses. Female dummy attractiveness scores were calculated as twice the sum of the occurrences of male behaviors B2 and B3, plus the sum of the occurrences of behavior B5. Behaviors B2 and B3 were assigned greater weight due to their stronger association with male arousal, as they were more consistently observed in our prior behavioral observations compared to behavior B5 (Table S1). Behavior B4 was excluded from the analysis, as males were unable to insert their genital capsule into the female dummies. We compared the attractiveness scores of the dummies using a paired t-test, following an assessment of normality with the Shapiro-Wilk test in R (version 3.5.0, R Core Team 2025). We additionally conducted a post hoc power analysis using the R package *pwr* (version 1.3-0) (Champely et al. 2020).

### CHC Profile Analysis

We extracted the hydrocarbons and analyzed 149 CHC profiles of *O. spinipes* wasps of different age classes (1 [= 1d], 3 [= 3d], 8 [= 8d], 14 [= 14d] days after eclosion) and representing the two sexes and the two female chemotypes (male [= m], female chemotype 1 [= c1], female chemotype 2 [= c2]). Sample sizes were as follows: N_c1,0d_ = 9; N_c1,1d_ = 7; N_c1,3d_ = 7; N_c1,8d_ = 24; N_c1,14d_ = 8; N_c2,0d_ = 3; N_c2,1d_ = 9; N_c2,3d_ = 9; N_c2,8d_ = 24; N_c2,14d_ = 9; N_m,0d_ = 8; N_m,3d_ = 10; N_m,8d_ = 19; N_m,14d_ = 3: Table S6). In total we analyzed profiles from 40 females of chemotype 1, 36 females of chemotype 2, and 40 males since CHCs from female wasps collected in 2016 were sampled multiple times: 2–3, 7–8, and 10–14 days post eclosion using a solid-phase microextraction (SPME) fiber (Supelco, coating: polydimethylsiloxane, 100 μm; Sigma Aldrich, Bellefonte, PA, USA). One female of chemotype 1 was not sampled at 1-day-old because of time constraints due to multiple runs with the GCMS. To facilitate handling, wasps sampled with SPME were anesthetized with CO_2_, and their metasoma was gently scrubbed with a SPME fiber for 2 min. This ensured the collection of a sufficient amount of CHCs. Using the SPME fiber allowed us to analyze the CHC profile of the same wasps at different ages, maximizing the use of our samples.

CHCs from wasps collected in 2018 and 2019 were sampled at day 0, day 3, and day 8 after eclosion. These wasps were killed by immersion in hexane for 2 min. in glass vials (Agilent Technologies, Santa Clara, CA, USA; screw-cap vials, 1.5 ml). The extracts were concentrated with a gentle stream of nitrogen to approximately 200 µL and then transferred to an insert vial (Agilent Technologies, Santa Clara, CA, USA; 250 ml glass with polymer feet). This extraction method was chosen to collect CHC extracts, which could later be applied to dead dummies for the bioassays.

CHC extracts on SPME fibers from wasps collected in 2016 were processed on an Agilent 6890 gas chromatograph coupled to an Agilent 5975 mass-selective detector (Agilent Technologies, Waldbronn, Germany). The GC was equipped with a DB-5 fused silica capillary column (30 m × 0.25 mm ID; df = 0.25 μm; J&W Scientific, Folsom, USA). The injector temperature was 250 °C. The following temperature profile was used: initial temperature of 60 °C, temperature increase of 20 °C per minute up to 150 °C, followed by a temperature increase of 5 °C per minute up to 300 °C, which was maintained for 10 min. CHC extracts in hexane solvent from wasps collected in 2018 and 2019 were analyzed using an Agilent 7890B gas chromatograph system (Agilent Technologies, Santa Clara, CA, USA) coupled to a 5977B mass-selective detector (Agilent Technologies, Santa Clara, CA, USA). The GC was equipped with a DB-5 column (30 m × 0.25 mm ID; df = 0.25 μm; J & W Scientific, Folsom, USA). The injector temperature was 250 °C. The following temperature profile was used: initial temperature of 40 °C, temperature increase of 10 °C per minute up to 300 °C, which was maintained for 10 min. Both systems used helium as the carrier gas at a constant flow rate of 1 mL/min. Electron ionization mass spectra (EI-MS) were acquired at an ionization voltage of 70 eV (source temperature: 230 °C). A standard solution of n-alkanes (C21–40) was injected weekly to control the sensitivity of the GC-MS and to provide a basis for the calculation of retention indices (RI).

Raw GC-MS data were analyzed using the MSD Enhanced ChemStation F.01.03.2357 software for Windows (Agilent Technologies, Böblingen, Germany). Individual compounds were characterized based on their diagnostic ions and their calculated Kovats retention index (Kovats 1958; Table S3). The abundance of each CHC was normalized by dividing it by the total abundance of all CHCs. Identification of some methyl-branched alkanes was not possible because they were not properly separated on the above instruments with the above settings and were therefore treated as a mixture. The identified hydrocarbons and their average normalized abundance in the profiles of male and female wasps of different ages are listed in Table S4.

We only considered CHCs with a relative abundance of at least 0.3% in the CHC profile. To counteract the effects of normalization that created compositional data, we CLR-transformed the relative abundance data in the R package *compositions* (version 1.40-2) (Aitchison et al. 2000; van den Boogaart and Tolosana-Delgado 2008). We used non-metric multidimensional scaling (NMDS) and principal component analysis (PCA) to visualize the data in two dimensions. NMDS plot was performed using the 149 CHC profile analyzed to have a global view at the distribution of the different CHC profiles. Boxplots of the different CHCs were also carried using the 149 CHC profiles.

PCA was used to identify CHCs that differed most between 0-day-old and 3-day-old female wasps, regardless of their chemotype. For this purpose, we analyzed the CHC profile data of each chemotype separately and identified CHCs that occurred in at least one sample with a relative abundance of at least 1% and at the same time were highly correlated (≥ 0.7) in their variation across all samples in the PCA along the first principal component (axis 1). For these analyses, we used only CHC profiles of wasps sampled in 2019 to minimize the risk of detecting differences caused by variations in GC/MS systems or extraction methods (SPME vs. hexane) rather than age.

NMDS plots were derived from Bray-Curtis distances between CHC profiles using the R packages *vegan* (version 2.4-2) (Oksanen 2011), *reshape2* (version 1.4.3) (Wickham 2007), and *dendextend* (version 1.5.2) (Galili 2015). PCAs were performed on the CLR-transformed abundance data using the R packages *FactoMineR* (Husson et al. 2017) and *Factoextra* (version 1.0.6) (Kassambara 2016).

Differences in the relative abundance of the identified CHCs between females of different age classes were assessed using a Kruskall-Wallis test with Dunn’s post-hoc comparisons applying the Bonferroni correction. The corresponding boxplots were drawn and statistical tests were performed in R (version 3.5.0; R Core Team 2025) using the package *ggplot2* (version 3.2.1) (Wickham 2016).

## Electronic Supplementary Material

Below is the link to the electronic supplementary material.

Supplementary Material 1

Supplementary Material 2

## Data Availability

Data supporting the findings of this study are available within the paper and its Supplementary Information files. Raw data files in another format are available from the corresponding author upon reasonable request.
