# Supplementary material for: Recognition of a Mating Partner Using Cuticular Hydrocarbons in a Species with an Extreme Intra-sexual Dimorphism
Source: J Chem Ecol. 2025 Jul 17;51(4):75. doi: 10.1007/s10886-025-01624-z (PMC12270971; doi:10.1007/s10886-025-01624-z)
Supplement: Supplementary file 1 — Supplementary Material 1 [file 10886_2025_1624_MOESM1_ESM.docx]

**Table S1**: Occurrences of individual *Odynerus spinipes* male mating behaviors (B1–5) described in detail in the results text. (B1) Male making antennal contact with the female, (B2) male mounting the female and wraps its antennae around the female’s antennae, (B3) male on top of the female extending its aedeagus and rubs it against the female’s metasoma, (B4) male inserting its genital capsule into the female’s genital opening, (B5) male lifting its metasoma and extruding its genital capsule after mating or unsuccessful mating attempts. d = day(s).

| **Male** | **Age** | **Female** | **Chemotype** | **Age** | **B1** | **B2** | **B3** | **B4** | **B5** |
| --- | --- | --- | --- | --- | --- | --- | --- | --- | --- |
| #12922 | 5 d | #12921 | 2 | 5 d | + | + | + | + | + |
| #12924 | 5 d | #12923 | 2 | 5 d | + | + | + | – | – |
| #12926 | 4 d | #12913 | 1 | 4 d | + | + | + | – | + |
| #12928 | 4 d | #12915 | 2 | 4 d | + | + | + | + | + |
| #12932 | 4 d | #12919 | 1 | 4 d | + | + | + | + | + |
| #12930 | 4 d | #12917 | 1 | 4 d | + | + | + | + | + |
| #12936 | 5 d | #12927 | 1 | 4 d | + | + | + | + | + |
| #12934 | 5 d | #12925 | 1 | 4 d | + | + | + | + | + |
| #12938 | 5 d | #12933 | 1 | 4 d | + | + | + | + | + |

**Table S2**: Samples used and behaviors recorded in the behavioral bioassays with female dummies coated with CHC extracts from 0-day- and 3-day-old females. d = day(s).

| Male CHC ID | Male ID |  | Female CHC ID | Female age | Chemotype | Female presented | Mounting attempt (count) | Gaster lifted (count) | Aedeagus extrusion (count) | Attractiveness score |
| --- | --- | --- | --- | --- | --- | --- | --- | --- | --- | --- |
| 12942 | 1 |  | 12893 | 0 d | 1 | first | 1 | 0 | 0 | 2 |
| 12942 | 1 |  | 12951 | 3 d | 1 | second | 2 | 2 | 1 | 8 |
| 12944 | 2 |  | 12949 | 3 d | 1 | first | 2 | 1 | 0 | 5 |
| 12944 | 2 |  | 12891 | 0 d | 1 | second | 1 | 0 | 0 | 2 |
| 12940 | 3 |  | 12895 | 0 d | 1 | first | 1 | 0 | 0 | 2 |
| 12940 | 3 |  | 12953 | 3 d | 1 | second | 1 | 2 | 1 | 6 |
| 12952 | 4 |  | 12899 | 0 d | 1 | first | 3 | 0 | 0 | 6 |
| 12952 | 4 |  | 12969 | 3 d | 1 | second | 3 | 4 | 0 | 10 |
| 12954 | 5 |  | 12931 | 3 d | 2 | first | 3 | 0 | 0 | 6 |
| 12954 | 5 |  | 12909 | 0 d | 2 | second | 0 | 0 | 0 | 0 |
| 12958 | 6 |  | 12907 | 0 d | 1 | first | 2 | 1 | 2 | 9 |
| 12958 | 6 |  | 12975 | 3 d | 1 | second | 2 | 1 | 1 | 7 |

**Table S3**: Retention indices and diagnostic ions of cuticular hydrocarbon compounds in extracts of *O. spinipes* males and females.

| **Compound** | **RI** | **Diagnostic ions** |
| --- | --- | --- |
| C21 | 2100 | 43, 57, 71, 85, 99, 113, 296 |
| 11-MeC21 | 2137 | 43, 57, 71, 85, 99, 113, 169, 310 |
| xC22ene | 2172 | 43, 55, 57, 69, 83, 97, 111, 125, 308 |
| yC22ene | 2180 | 43, 55, 57, 69, 83, 97, 111, 125, 308 |
| C22 | 2200 | 43, 57, 71, 85, 99, 113, 310 |
| 11-MeC22 | 2236 | 43, 57, 71, 85, 99, 113, 169, 183, 324 |
| 10-MeC22 | 2256 | 43, 57, 71, 85, 99, 113, 155, 197, 324 |
| x,yC23diene | 2267 | 44, 55, 69, 82, 96, 109, 124, 320 |
| 10C23ene | 2270 | 43, 55, 57, 69, 83, 97, 111, 125, 322 |
| 9C23ene | 2272 | 43, 55, 57, 69, 83, 97, 111, 125, 322 |
| 8C23ene | 2275 | 43, 55, 57, 69, 83, 97, 111, 125, 322 |
| 7C23ene | 2279 | 43, 55, 57, 69, 83, 97, 111, 125, 322 |
| xC23ene | 2286 | 43, 55, 57, 69, 83, 97, 111, 125, 322 |
| 5C23ene | 2289 | 43, 55, 57, 69, 83, 97, 111, 125, 322 |
| yC23ene | 2294 | 43, 55, 57, 69, 83, 97, 111, 125, 322 |
| C23 | 2300 | 43, 57, 71, 85, 99, 113, 324 |
| 11-; 9-MeC23 | 2334 | 43, 57, 71, 85, 99, 113, 141, 169, 197, 225, 338 |
| 7-MeC23 | 2340 | 43, 57, 71, 85, 99, 113, 253, 338 |
| 5-MeC23 | 2349 | 43, 57, 71, 85, 99, 113, 281, 338 |
| 9,13-diMeC23 | 2363 | 43, 57, 71, 85, 99, 113, 141, 169, 211, 239, 352 |
| 3-MeC23 | 2372 | 43, 57, 71, 85, 99, 113, 309, 338 |
| 11C24ene | 2373 | 43, 55, 57, 69, 83, 97, 111, 125, 336 |
| 9C24ene | 2381 | 43, 55, 57, 69, 83, 97, 111, 125, 336 |
| C24 | 2400 | 43, 57, 71, 85, 99, 113, 338 |
| 11-MeC24 | 2406 | 43, 57, 71, 85, 99, 113, 169, 211, 352 |
| x,y-diMeC24 | 2434 | 44, 55, 69, 82, 96, 109, 124, 348 |
| x,yC25diene/5-MeC24 | 2450 | 44, 55, 69, 82, 96, 109, 124, 348/43, 57, 71, 85, 99, 113, 295, 352 |
| z,aC25diene | 2458 | 44, 55, 69, 82, 96, 109, 124, 348 |
| 4-MeC24 | 2463 | 43, 57, 71, 85, 99, 113, 309, 352 |
| 10C25ene | 2473 | 43, 55, 57, 69, 83, 97, 111, 125, 350 |
| 9C25ene | 2475 | 43, 55, 57, 69, 83, 97, 111, 125, 350 |
| 8C25ene | 2478 | 43, 55, 57, 69, 83, 97, 111, 125, 350 |
| 7C25ene | 2483 | 43, 55, 57, 69, 83, 97, 111, 125, 350 |
| xC25ene | 2486 | 43, 55, 57, 69, 83, 97, 111, 125, 350 |
| 5C25ene | 2494 | 43, 55, 57, 69, 83, 97, 111, 125, 350 |
| C25 | 2500 | 43, 57, 71, 85, 99, 113, 352 |
| X1 | 2519 |  |
| 13-; 11-; 9-MeC25 | 2533 | 43, 57, 71, 85, 99, 113, 141, 169, 197, 225, 253, 366 |
| 7-MeC25 | 2540 | 43, 57, 71, 85, 99, 113, 281, 366 |
| 5-MeC25 | 2549 | 43, 57, 71, 85, 99, 113, 323, 366 |
| 11,15-diMeC25 | 2562 | 43, 57, 71, 85, 99, 113, 169, 239, 380 |
| 9,17-diMeC25 | 2569 | 43, 57, 71, 85, 99, 113, 141, 267, 380 |
| 3-MeC25 | 2573 | 43, 57, 71, 85, 99, 113, 337, 366 |
| 12C26ene | 2582 | 43, 55, 57, 69, 83, 97, 111, 125, 364 |
| 11C26ene/5,11-/5,13-diMeC25 | 2582 | 43, 55, 57, 69, 83, 97, 111, 125, 350/43, 57, 71, 85, 99, 113, 183, 197, 211, 225, 323, 380 |
| C26 | 2600 | 43, 57, 71, 85, 99, 113, 366 |
| 11-MeC26 | 2605 | 43, 57, 71, 85, 99, 113, 169, 239, 380 |
| 10-MeC26 | 2637 | 43, 57, 71, 85, 99, 113, 155, 253, 380 |
| 7-MeC26 | 2638 | 43, 57, 71, 85, 99, 113, 295, 380 |
| x2 | 2644 |  |
| x,yC27diene | 2654 | 44, 55, 69, 82, 96, 109, 124, 376 |
| z,aC27diene | 2663 | 44, 55, 69, 82, 96, 109, 124, 376 |
| xC27ene | 2668 | 43, 55, 57, 69, 83, 97, 111, 125, 378 |
| 12C27ene | 2672 | 43, 55, 57, 69, 83, 97, 111, 125, 378 |
| 10C27ene | 2675 | 43, 55, 57, 69, 83, 97, 111, 125, 378 |
| 9C27ene | 2676 | 43, 55, 57, 69, 83, 97, 111, 125, 378 |
| 8C27ene | 2682 | 43, 55, 57, 69, 83, 97, 111, 125, 378 |
| 7C27ene | 2684 | 43, 55, 57, 69, 83, 97, 111, 125, 378 |
| 5C27ene | 2693 | 43, 55, 57, 69, 83, 97, 111, 125, 378 |
| C27 | 2700 | 43, 57, 71, 85, 99, 113, 380 |
| 11-; 9-MeC27 | 2733 | 43, 57, 71, 85, 99, 113, 141, 169, 253, 281, 394 |
| 7-MeC27 | 2739 | 43, 57, 71, 85, 99, 113, 309, 394 |
| 5-MeC27 | 2749 | 43, 57, 71, 85, 99, 113, 337, 394 |
| xC28ene/x-MeC27 | 2768 | 43, 55, 57, 69, 83, 97, 111, 125, 392 |
| yC28ene/3-MeC27 | 2776 | 43, 55, 57, 69, 83, 97, 111, 125, 392/43, 57, 71, 85, 99, 113, 365, 394 |
| 9C28ene | 2784 | 43, 55, 57, 69, 83, 97, 111, 125, 392 |
| C28 | 2800 | 43, 57, 71, 85, 99, 113, 394 |
| 9-MeC28 | 2836 | 43, 57, 71, 85, 99, 113, 141, 295,408 |
| x,yC29diene | 2853 | 44, 55, 69, 82, 96, 109, 124, 404 |
| z,aC29diene | 2858 | 44, 55, 69, 82, 96, 109, 124, 404 |
| 14; 12C29ene | 2869 | 43, 55, 57, 69, 83, 97, 111, 125, 406 |
| 10C29ene | 2876 | 43, 55, 57, 69, 83, 97, 111, 125, 406 |
| 9C29ene | 2877 | 43, 55, 57, 69, 83, 97, 111, 125, 406 |
| 8C29ene | 2884 | 43, 55, 57, 69, 83, 97, 111, 125, 406 |
| 7C29ene | 2884 | 43, 55, 57, 69, 83, 97, 111, 125, 406 |
| C29 | 2900 | 43, 57, 71, 85, 99, 113, 408 |
| 11-; 9-MeC29 | 2935 | 43, 57, 71, 85, 99, 113, 141, 169, 281, 309, 422 |
| 5-MeC29 | 2950 | 43, 57, 71, 85, 99, 113, 365, 422 |
| 9,19-diMeC29 | 2966 | 43, 57, 71, 85, 99, 113, 141, 169, 295, 323, 436 |
| 5,x-diMeC29 | 2974 | 43, 57, 71, 85, 99, 113, 379, 436 |
| C30 | 300 | 43, 57, 71, 85, 99, 113, 422 |
| 11-; 10-MeC30 | 3032 | 43, 57, 71, 85, 99, 113, 155, 169, 295, 309, 436 |
| x,yC31diene | 3049 | 44, 55, 69, 82, 96, 109, 124, 432 |
| x3 | 3057 |  |
| xC31ene | 3070 | 43, 55, 57, 69, 83, 97, 111, 125, 434 |
| yC31ene | 3078 | 43, 55, 57, 69, 83, 97, 111, 125, 434 |
| zC31ene | 3086 | 43, 55, 57, 69, 83, 97, 111, 125, 434 |
| C31 | 3100 | 43, 57, 71, 85, 99, 113, 436 |
| 11-MeC31 | 3117 | 43, 57, 71, 85, 99, 113, 169, 309, 450 |
| xC33ene | 3258 | 43, 55, 57, 69, 83, 97, 111, 125, 462 |
| C33 | 3300 | 43, 57, 71, 85, 99, 113, 464 |
| 13-; 11-MeC33 | 3322 | 43, 57, 71, 85, 99, 113, 169, 197, 309, 337, 478 |
| x-MeC33 | 3351 |  |
| 11,21-diMeC33 | 3372 | 43, 57, 71, 85, 99, 113, 169, 211, 323, 365, 506 |

**Table S4**: Relative abundances (% ± s.e.) of cuticular hydrocarbon compounds in extracts of *O. spinipes* males and females (c1 = chemotype 1; c2 = chemotype 2) on the day of eclosion and 3 days after eclosion.

| **Compound** | **0-day-old males** | | | **0-day-old females (c1)** | | | **0-day-old females (c2)** | | | **3-day-old males** | | | **3-day-old females (c1)** | | | **3-day-old females (c2)** | | |
| --- | --- | --- | --- | --- | --- | --- | --- | --- | --- | --- | --- | --- | --- | --- | --- | --- | --- | --- |
| C21 | 1.33 | ± | 0.25 | 2.54 | ± | 0.40 | 2.30 | ± | 0.45 | 0.44 | ± | 0.11 | 0.42 | ± | 0.17 | 0.62 | ± | 0.33 |
| 11-MeC21 | 0.04 | ± | 0.02 | 0.05 | ± | 0.02 | 0.04 | ± | 0.01 | 0.01 | ± | 0.01 | 0.01 | ± | 0.01 | 0.00 | ± | 0.00 |
| xC22ene | 0.01 | ± | 0.01 | 0.00 | ± | 0.01 | 0.00 | ± | 0.00 | 0.01 | ± | 0.01 | 0.02 | ± | 0.01 | 0.01 | ± | 0.02 |
| yC22ene | 0.00 | ± | 0.01 | 0.01 | ± | 0.01 | 0.00 | ± | 0.00 | 0.04 | ± | 0.05 | 0.07 | ± | 0.08 | 0.01 | ± | 0.01 |
| C22 | 0.40 | ± | 0.12 | 0.65 | ± | 0.16 | 0.50 | ± | 0.17 | 0.18 | ± | 0.04 | 0.31 | ± | 0.04 | 0.23 | ± | 0.08 |
| 11-MeC22 | 0.00 | ± | 0.00 | 0.00 | ± | 0.00 | 0.01 | ± | 0.02 | 0.02 | ± | 0.02 | 0.01 | ± | 0.01 | 0.00 | ± | 0.00 |
| 10-MeC22 | 0.03 | ± | 0.01 | 0.02 | ± | 0.02 | 0.00 | ± | 0.00 | 0.01 | ± | 0.02 | 0.02 | ± | 0.02 | 0.01 | ± | 0.01 |
| x,yC23diene | 0.01 | ± | 0.01 | 0.01 | ± | 0.01 | 0.00 | ± | 0.00 | 0.01 | ± | 0.02 | 0.00 | ± | 0.01 | 0.00 | ± | 0.00 |
| 10C23ene | 0.00 | ± | 0.00 | 0.00 | ± | 0.00 | 0.97 | ± | 0.34 | 0.00 | ± | 0.00 | 0.00 | ± | 0.00 | 0.80 | ± | 1.32 |
| 9C23ene | 2.10 | ± | 0.67 | 0.66 | ± | 0.15 | 0.00 | ± | 0.00 | 1.33 | ± | 0.48 | 2.41 | ± | 1.37 | 0.00 | ± | 0.00 |
| 8C23ene | 0.00 | ± | 0.00 | 0.00 | ± | 0.00 | 1.46 | ± | 0.86 | 0.00 | ± | 0.00 | 0.00 | ± | 0.00 | 2.92 | ± | 2.17 |
| 7C23ene | 2.87 | ± | 0.76 | 2.45 | ± | 1.60 | 0.00 | ± | 0.00 | 5.92 | ± | 1.89 | 6.40 | ± | 4.57 | 0.12 | ± | 0.25 |
| xC23ene | 0.02 | ± | 0.06 | 0.00 | ± | 0.00 | 0.00 | ± | 0.00 | 0.00 | ± | 0.00 | 0.00 | ± | 0.00 | 0.00 | ± | 0.00 |
| 5C23ene | 0.03 | ± | 0.02 | 0.09 | ± | 0.03 | 0.00 | ± | 0.00 | 0.10 | ± | 0.05 | 0.71 | ± | 0.31 | 0.00 | ± | 0.00 |
| yC23ene | 0.00 | ± | 0.00 | 0.02 | ± | 0.02 | 0.01 | ± | 0.02 | 0.00 | ± | 0.00 | 0.02 | ± | 0.03 | 0.01 | ± | 0.02 |
| C23 | 11.1 | ± | 1.90 | 14.9 | ± | 1.75 | 12.5 | ± | 2.84 | 6.26 | ± | 0.27 | 11.6 | ± | 1.39 | 9.32 | ± | 1.36 |
| 11-; 9-MeC23 | 2.83 | ± | 0.44 | 2.49 | ± | 0.34 | 2.03 | ± | 0.42 | 2.01 | ± | 0.38 | 0.52 | ± | 0.24 | 0.27 | ± | 0.16 |
| 7-MeC23 | 0.18 | ± | 0.03 | 0.04 | ± | 0.06 | 0.02 | ± | 0.04 | 0.13 | ± | 0.06 | 0.01 | ± | 0.02 | 0.00 | ± | 0.01 |
| 5-MeC23 | 0.21 | ± | 0.04 | 0.24 | ± | 0.04 | 0.17 | ± | 0.06 | 0.10 | ± | 0.02 | 0.08 | ± | 0.03 | 0.03 | ± | 0.07 |
| 9,13-diMeC23 | 0.06 | ± | 0.02 | 0.12 | ± | 0.03 | 0.21 | ± | 0.16 | 0.00 | ± | 0.00 | 0.01 | ± | 0.02 | 0.05 | ± | 0.10 |
| 3-MeC23 | 1.10 | ± | 0.14 | 0.63 | ± | 0.13 | 0.30 | ± | 0.28 | 1.22 | ± | 0.33 | 0.94 | ± | 0.44 | 0.16 | ± | 0.16 |
| 11C24ene | 0.00 | ± | 0.00 | 0.00 | ± | 0.00 | 0.00 | ± | 0.00 | 0.00 | ± | 0.00 | 0.00 | ± | 0.00 | 0.08 | ± | 0.11 |
| 9C24ene | 0.30 | ± | 0.07 | 0.26 | ± | 0.10 | 0.10 | ± | 0.02 | 0.65 | ± | 0.22 | 0.63 | ± | 0.54 | 0.09 | ± | 0.07 |
| C24 | 0.51 | ± | 0.07 | 0.83 | ± | 0.18 | 0.59 | ± | 0.14 | 0.40 | ± | 0.08 | 0.50 | ± | 0.09 | 0.31 | ± | 0.07 |
| 11-MeC24 | 0.12 | ± | 0.03 | 0.14 | ± | 0.03 | 0.12 | ± | 0.04 | 0.09 | ± | 0.02 | 0.02 | ± | 0.02 | 0.01 | ± | 0.01 |
| x,y-diMeC24 | 0.22 | ± | 0.06 | 0.21 | ± | 0.07 | 0.18 | ± | 0.04 | 0.22 | ± | 0.04 | 0.02 | ± | 0.03 | 0.05 | ± | 0.11 |
| x,yC25diene/ 5-MeC24 | 0.07 | ± | 0.07 | 0.06 | ± | 0.04 | 0.05 | ± | 0.02 | 0.01 | ± | 0.02 | 0.02 | ± | 0.02 | 0.06 | ± | 0.11 |
| z,aC25diene | 0.07 | ± | 0.08 | 0.04 | ± | 0.05 | 0.05 | ± | 0.01 | 0.05 | ± | 0.02 | 0.04 | ± | 0.05 | 0.09 | ± | 0.13 |
| 4-MeC24 | 0.03 | ± | 0.06 | 0.03 | ± | 0.04 | 0.04 | ± | 0.04 | 0.02 | ± | 0.03 | 0.00 | ± | 0.01 | 0.00 | ± | 0.00 |
| 10C25ene | 0.00 | ± | 0.00 | 0.00 | ± | 0.00 | 3.62 | ± | 0.75 | 0.00 | ± | 0.00 | 0.00 | ± | 0.00 | 6.63 | ± | 1.63 |
| 9C25ene | 9.60 | ± | 1.61 | 8.07 | ± | 3.05 | 0.00 | ± | 0.00 | 11.3 | ± | 1.60 | 11.6 | ± | 8.23 | 0.00 | ± | 0.00 |
| 8C25ene | 0.00 | ± | 0.00 | 0.00 | ± | 0.00 | 1.78 | ± | 0.63 | 0.00 | ± | 0.00 | 0.00 | ± | 0.00 | 4.06 | ± | 0.74 |
| 7C25ene | 11.1 | ± | 2.02 | 9.25 | ± | 4.31 | 0.32 | ± | 0.55 | 14.2 | ± | 1.34 | 15 | ± | 7.36 | 0.00 | ± | 0.00 |
| xC25ene | 0.00 | ± | 0.00 | 0.02 | ± | 0.06 | 0.00 | ± | 0.00 | 0.00 | ± | 0.00 | 1.35 | ± | 2.67 | 0.00 | ± | 0.00 |
| 5C25ene | 0.00 | ± | 0.00 | 0.00 | ± | 0.00 | 0.00 | ± | 0.00 | 0.32 | ± | 0.10 | 0.65 | ± | 0.26 | 0.00 | ± | 0.00 |
| C25 | 7.60 | ± | 0.86 | 9.65 | ± | 0.80 | 7.86 | ± | 0.96 | 7.03 | ± | 0.62 | 7.94 | ± | 0.85 | 6.32 | ± | 0.76 |
| X1 | 0.00 | ± | 0.00 | 0.00 | ± | 0.00 | 0.00 | ± | 0.00 | 0.00 | ± | 0.00 | 0.13 | ± | 0.18 | 0.00 | ± | 0.00 |
| 13-; 11-; 9-MeC25 | 3.96 | ± | 0.36 | 2.71 | ± | 0.47 | 2.30 | ± | 0.34 | 4.46 | ± | 0.28 | 0.25 | ± | 0.28 | 0.41 | ± | 0.50 |
| 7-MeC25 | 0.42 | ± | 0.09 | 0.41 | ± | 0.18 | 0.56 | ± | 0.36 | 0.21 | ± | 0.20 | 0.07 | ± | 0.05 | 0.05 | ± | 0.07 |
| 5-MeC25 | 0.88 | ± | 0.35 | 1.25 | ± | 0.44 | 1.08 | ± | 0.25 | 0.37 | ± | 0.08 | 0.20 | ± | 0.09 | 0.13 | ± | 0.12 |
| 11,15-diMeC25 | 0.17 | ± | 0.06 | 0.24 | ± | 0.07 | 0.19 | ± | 0.07 | 0.00 | ± | 0.00 | 0.02 | ± | 0.02 | 0.01 | ± | 0.02 |
| 9,17-diMeC25 | 0.00 | ± | 0.00 | 0.07 | ± | 0.04 | 0.09 | ± | 0.16 | 0.02 | ± | 0.04 | 0.06 | ± | 0.04 | 0.33 | ± | 0.36 |
| 3-MeC25 | 1.16 | ± | 0.18 | 0.77 | ± | 0.16 | 0.70 | ± | 0.23 | 1.81 | ± | 0.35 | 0.91 | ± | 0.42 | 0.51 | ± | 0.33 |
| 12C26ene | 0.06 | ± | 0.08 | 0.75 | ± | 0.15 | 0.00 | ± | 0.00 | 0.21 | ± | 0.10 | 0.39 | ± | 0.25 | 0.06 | ± | 0.14 |
| 11C26ene/ 5,11-/5,13-diMeC25 | 0.64 | ± | 0.18 | 0.00 | ± | 0.00 | 0.49 | ± | 0.10 | 0.66 | ± | 0.13 | 0.02 | ± | 0.02 | 0.27 | ± | 0.10 |
| C26 | 0.41 | ± | 0.09 | 0.62 | ± | 0.09 | 0.51 | ± | 0.07 | 0.39 | ± | 0.06 | 0.49 | ± | 0.06 | 0.37 | ± | 0.26 |
| 11-MeC26 | 0.19 | ± | 0.09 | 0.07 | ± | 0.11 | 0.10 | ± | 0.10 | 0.45 | ± | 0.13 | 0.00 | ± | 0.00 | 0.00 | ± | 0.00 |
| 10-MeC26 | 0.11 | ± | 0.07 | 0.05 | ± | 0.03 | 0.04 | ± | 0.04 | 0.10 | ± | 0.02 | 0.02 | ± | 0.01 | 0.01 | ± | 0.01 |
| 7-MeC26 | 0.00 | ± | 0.00 | 0.00 | ± | 0.00 | 0.11 | ± | 0.06 | 0.00 | ± | 0.00 | 0.00 | ± | 0.00 | 0.01 | ± | 0.01 |
| x2 | 0.00 | ± | 0.00 | 0.00 | ± | 0.00 | 0.17 | ± | 0.07 | 0.00 | ± | 0.00 | 0.00 | ± | 0.00 | 0.13 | ± | 0.14 |
| x,yC27diene | 0.33 | ± | 0.33 | 0.17 | ± | 0.16 | 0.27 | ± | 0.03 | 0.21 | ± | 0.06 | 0.09 | ± | 0.08 | 0.30 | ± | 0.35 |
| z,aC27diene | 0.27 | ± | 0.22 | 0.19 | ± | 0.18 | 0.00 | ± | 0.00 | 0.25 | ± | 0.05 | 0.04 | ± | 0.04 | 0.48 | ± | 0.27 |
| xC27ene | 0.08 | ± | 0.23 | 0.00 | ± | 0.00 | 0.00 | ± | 0.00 | 0.04 | ± | 0.10 | 0.00 | ± | 0.01 | 0.00 | ± | 0.00 |
| 12C27ene | 0.00 | ± | 0.00 | 0.00 | ± | 0.00 | 6.52 | ± | 0.72 | 0.00 | ± | 0.00 | 0.00 | ± | 0.00 | 13.87 | ± | 3.22 |
| 10C27ene | 0.00 | ± | 0.00 | 0.00 | ± | 0.00 | 3.33 | ± | 0.44 | 0.00 | ± | 0.00 | 0.00 | ± | 0.00 | 2.42 | ± | 2.61 |
| 9C27ene | 12.2 | ± | 0.64 | 10.5 | ± | 4.18 | 0.00 | ± | 0.00 | 10.6 | ± | 1.28 | 13.1 | ± | 6.47 | 0.00 | ± | 0.00 |
| 8C27ene | 0.00 | ± | 0.00 | 0.00 | ± | 0.00 | 4.76 | ± | 0.75 | 0.00 | ± | 0.00 | 0.00 | ± | 0.00 | 7.27 | ± | 1.01 |
| 7C27ene | 6.80 | ± | 1.10 | 5.76 | ± | 2.10 | 0.00 | ± | 0.00 | 7.65 | ± | 1.37 | 4.97 | ± | 2.49 | 0.00 | ± | 0.00 |
| 5C27ene | 0.23 | ± | 0.11 | 0.39 | ± | 0.05 | 0.00 | ± | 0.00 | 0.57 | ± | 0.09 | 1.13 | ± | 0.30 | 0.00 | ± | 0.00 |
| C27 | 7.97 | ± | 1.27 | 9.40 | ± | 0.87 | 8.05 | ± | 0.42 | 7.40 | ± | 1.29 | 8.08 | ± | 0.86 | 6.37 | ± | 0.89 |
| 11-; 9-MeC27 | 1.29 | ± | 0.30 | 0.63 | ± | 0.18 | 0.55 | ± | 0.14 | 1.56 | ± | 0.33 | 0.65 | ± | 0.49 | 0.40 | ± | 0.32 |
| 7-MeC27 | 0.22 | ± | 0.08 | 0.88 | ± | 0.23 | 0.75 | ± | 0.11 | 0.13 | ± | 0.06 | 0.07 | ± | 0.09 | 0.05 | ± | 0.08 |
| 5-MeC27 | 0.00 | ± | 0.00 | 0.40 | ± | 0.10 | 0.32 | ± | 0.07 | 0.00 | ± | 0.00 | 0.10 | ± | 0.02 | 0.07 | ± | 0.07 |
| xC28ene/x-MeC27 | 0.00 | ± | 0.00 | 0.09 | ± | 0.15 | 0.26 | ± | 0.07 | 0.00 | ± | 0.00 | 0.03 | ± | 0.05 | 0.22 | ± | 0.26 |
| yC28ene/3-MeC27 | 1.41 | ± | 0.22 | 0.48 | ± | 0.23 | 0.91 | ± | 0.13 | 2.01 | ± | 0.15 | 0.33 | ± | 0.24 | 0.92 | ± | 0.21 |
| 9C28ene | 0.00 | ± | 0.00 | 0.00 | ± | 0.00 | 0.09 | ± | 0.15 | 0.06 | ± | 0.07 | 0.00 | ± | 0.00 | 0.09 | ± | 0.09 |
| C28 | 0.21 | ± | 0.03 | 0.27 | ± | 0.08 | 0.19 | ± | 0.03 | 0.31 | ± | 0.07 | 0.20 | ± | 0.06 | 0.13 | ± | 0.11 |
| 9-MeC28 | 0.00 | ± | 0.00 | 0.03 | ± | 0.03 | 0.01 | ± | 0.02 | 0.00 | ± | 0.00 | 0.00 | ± | 0.00 | 0.00 | ± | 0.00 |
| x,yC29diene | 0.13 | ± | 0.13 | 0.08 | ± | 0.12 | 1.00 | ± | 0.61 | 0.05 | ± | 0.02 | 0.02 | ± | 0.07 | 1.89 | ± | 0.59 |
| z,aC29diene | 0.30 | ± | 0.18 | 0.22 | ± | 0.33 | 0.58 | ± | 0.45 | 0.10 | ± | 0.10 | 0.04 | ± | 0.18 | 1.75 | ± | 0.86 |
| 14; 12C29ene | 0.00 | ± | 0.00 | 0.00 | ± | 0.00 | 3.54 | ± | 2.01 | 0.00 | ± | 0.00 | 0.00 | ± | 0.00 | 7.07 | ± | 2.04 |
| 10C29ene | 0.00 | ± | 0.00 | 0.00 | ± | 0.00 | 12.1 | ± | 1.26 | 0.00 | ± | 0.00 | 0.00 | ± | 0.00 | 13.9 | ± | 3.94 |
| 9C29ene | 4.12 | ± | 0.91 | 4.46 | ± | 3.19 | 0.00 | ± | 0.00 | 3.30 | ± | 0.83 | 3.93 | ± | 3.81 | 0.00 | ± | 0.00 |
| 8C29ene | 0.00 | ± | 0.00 | 0.00 | ± | 0.00 | 9.62 | ± | 1.13 | 0.00 | ± | 0.00 | 0.12 | ± | 0.13 | 4.07 | ± | 2.88 |
| 7C29ene | 0.64 | ± | 0.41 | 0.32 | ± | 0.40 | 0.00 | ± | 0.00 | 1.08 | ± | 0.21 | 0.37 | ± | 0.32 | 0.00 | ± | 0.00 |
| C29 | 2.28 | ± | 0.51 | 4.25 | ± | 0.94 | 3.79 | ± | 0.08 | 2.51 | ± | 0.73 | 2.87 | ± | 1.09 | 2.27 | ± | 0.39 |
| 11-; 9-MeC29 | 0.30 | ± | 0.12 | 0.55 | ± | 0.23 | 0.64 | ± | 0.19 | 0.16 | ± | 0.04 | 0.08 | ± | 0.09 | 0.07 | ± | 0.09 |
| 5-MeC29 | 0.00 | ± | 0.00 | 0.04 | ± | 0.06 | 0.09 | ± | 0.09 | 0.00 | ± | 0.00 | 0.00 | ± | 0.00 | 0.17 | ± | 0.12 |
| 9,19-diMeC29 | 0.02 | ± | 0.03 | 0.07 | ± | 0.06 | 0.10 | ± | 0.12 | 0.00 | ± | 0.00 | 0.00 | ± | 0.00 | 0.07 | ± | 0.08 |
| 5,x-diMeC29 | 0.15 | ± | 0.06 | 0.03 | ± | 0.04 | 0.04 | ± | 0.08 | 0.16 | ± | 0.03 | 0.00 | ± | 0.01 | 0.04 | ± | 0.07 |
| C30 | 0.01 | ± | 0.02 | 0.03 | ± | 0.03 | 0.00 | ± | 0.00 | 0.02 | ± | 0.02 | 0.01 | ± | 0.01 | 0.00 | ± | 0.00 |
| 11-; 10-MeC30 | 0.00 | ± | 0.01 | 0.02 | ± | 0.03 | 0.04 | ± | 0.07 | 0.00 | ± | 0.00 | 0.00 | ± | 0.00 | 0.00 | ± | 0.00 |
| x,yC31diene | 0.00 | ± | 0.00 | 0.00 | ± | 0.00 | 0.28 | ± | 0.24 | 0.00 | ± | 0.00 | 0.00 | ± | 0.00 | 0.40 | ± | 0.45 |
| x3 | 0.00 | ± | 0.00 | 0.00 | ± | 0.00 | 0.03 | ± | 0.05 | 0.00 | ± | 0.00 | 0.00 | ± | 0.00 | 0.32 | ± | 0.25 |
| xC31ene | 0.00 | ± | 0.00 | 0.01 | ± | 0.01 | 0.33 | ± | 0.22 | 0.00 | ± | 0.00 | 0.00 | ± | 0.00 | 0.60 | ± | 0.29 |
| yC31ene | 0.49 | ± | 0.19 | 0.18 | ± | 0.12 | 0.74 | ± | 0.27 | 0.28 | ± | 0.09 | 0.08 | ± | 0.08 | 0.43 | ± | 0.32 |
| zC31ene | 0.00 | ± | 0.00 | 0.01 | ± | 0.02 | 0.00 | ± | 0.00 | 0.08 | ± | 0.04 | 0.00 | ± | 0.00 | 0.00 | ± | 0.00 |
| C31 | 0.44 | ± | 0.12 | 0.36 | ± | 0.20 | 0.16 | ± | 0.06 | 0.31 | ± | 0.09 | 0.09 | ± | 0.04 | 0.14 | ± | 0.20 |
| 11-MeC31 | 0.34 | ± | 0.18 | 0.61 | ± | 0.26 | 0.49 | ± | 0.19 | 0.12 | ± | 0.04 | 0.06 | ± | 0.05 | 0.03 | ± | 0.05 |
| xC33ene | 0.28 | ± | 0.16 | 0.09 | ± | 0.07 | 0.09 | ± | 0.15 | 0.21 | ± | 0.05 | 0.01 | ± | 0.02 | 0.05 | ± | 0.07 |
| C33 | 0.21 | ± | 0.10 | 0.09 | ± | 0.08 | 0.20 | ± | 0.21 | 0.21 | ± | 0.05 | 0.02 | ± | 0.02 | 0.01 | ± | 0.02 |
| 13-; 11-MeC33 | 0.19 | ± | 0.11 | 0.39 | ± | 0.26 | 0.10 | ± | 0.07 | 0.05 | ± | 0.05 | 0.03 | ± | 0.02 | 0.02 | ± | 0.04 |
| x-MeC33 | 0.03 | ± | 0.03 | 0.23 | ± | 0.41 | 0.10 | ± | 0.08 | 0.00 | ± | 0.00 | 0.00 | ± | 0.00 | 0.02 | ± | 0.03 |
| 11,21-diMeC33 | 0.07 | ± | 0.05 | 0.12 | ± | 0.12 | 0.09 | ± | 0.09 | 0.00 | ± | 0.00 | 0.00 | ± | 0.00 | 0.01 | ± | 0.02 |

**Table S5**: P-values of tests comparing the relative abundances of methyl-branched alkanes, which were found in PCA analyses to decrease in females of chemotype 1 (= c1) and chemotype 2 (= c2), but not in males. P-values were Bonferroni-corrected for multiple testing within each group.

|  |  | **Females c1** | | **Females c2** | | **Males** | |
| --- | --- | --- | --- | --- | --- | --- | --- |
| Compound | Comparison | p | adjusted p | p | adjusted p | p | adjusted p |
| sum of methyl branched | 0d-1d | 0.00214 | 0.03214 | 0.62488 | 1 |  |  |
| sum of methyl branched | 0d-3d | 0.00057 | 0.00854 | 0.00444 | 0.06654 | 0.18260 | 0.91301 |
| sum of methyl branched | 1d-3d | 0.72285 | 1 | 0.00127 | 0.01906 |  |  |
| 11-; 9-MeC23 | 0d-1d | 0.17956 | 1 | 1 | 1 |  |  |
| 11-; 9-MeC23 | 0d-3d | 9.96E-05 | 0.00149 | 0.01123 | 0.16845 | 0.00187 | 0.00935 |
| 11-; 9-MeC23 | 1d-3d | 0.01623 | 0.24343 | 0.00050 | 0.00756 |  |  |
| 7-MeC27 | 0d-1d | 3.77E-05 | 0.00057 | 0.00043 | 0.00641 |  |  |
| 7-MeC27 | 0d-3d | 0.00373 | 0.05595 | 0.02603 | 0.39040 | 0.01239 | 0.06196 |
| 7-MeC27 | 1d-3d | 0.24962 | 1 | 0.06373 | 0.95590 |  |  |
| 13-; 11-; 9-MeC25 | 0d-1d | 0.43414 | 1 | 0.35994 | 1 |  |  |
| 13-; 11-; 9-MeC25 | 0d-3d | 0.00020 | 0.00305 | 0.06298 | 0.94469 | 0.37426 | 1 |
| 13-; 11-; 9-MeC25 | 1d-3d | 0.00569 | 0.08533 | 0.00013 | 0.00195 |  |  |
| x,y-diMec24 | 0d-1d | 0.02113 | 0.31691 | 0.00236 | 0.03543 |  |  |
| x,y-diMec24 | 0d-3d | 9.80E-05 | 0.00147 | 0.05996 | 0.89940 | 0.24806 | 1 |
| x,y-diMec24 | 1d-3d | 0.13390 | 1 | 0.09786 | 1 |  |  |

**Table S6**: Number of CHC profiles analyzed for *O. spinipes* females of chemotype1 (= c1), of chemotype 2 (= c2), and males at specific ages (0 days, 1 day, 3 days, 8 days, and 14 days old), sampled in 2016, 2018, and 2019 from trap nests collected in Büchelberg.

|  | **2016** | **2018** | **2019** | **Total** |
| --- | --- | --- | --- | --- |
| **Females C1** |  |  |  | **55** |
| 0d |  |  | 9 | 9 |
| 1d | 7* |  |  | 7 |
| 3d |  |  | 7 | 7 |
| 8d | 8* |  | 16 | 24 |
| 14d | 8* |  |  | 8 |
| **Females C2** |  |  |  | **54** |
| 0d |  |  | 3 | 3 |
| 1d | 9* |  |  | 9 |
| 3d |  | 5 | 4 | 9 |
| 8d | 9* | 10 | 5 | 24 |
| 14d | 9* |  |  | 9 |
| **Males** |  |  |  | **40** |
| 0d |  |  | 8 | 8 |
| 3d |  | 3 | 7 | 10 |
| 8d |  | 3 | 16 | 19 |
| 14d |  | 3 |  | 3 |
| **Total** | **50** | **24** | **75** | **149** |

* Samples observed throughout their lifespan, with some having their hydrocarbons sampled three times. Seven females of chemotype 1 and nine females of chemotype 2 had their CHC profiles sampled three times. One female of chemotype 1 was sampled twice.
