# Supplementary material for: Recognition of a Mating Partner Using Cuticular Hydrocarbons in a Species with an Extreme Intra-sexual Dimorphism
Source: J Chem Ecol. 2025 Jul 17;51(4):75. doi: 10.1007/s10886-025-01624-z (PMC12270971; doi:10.1007/s10886-025-01624-z)
Supplement: Supplementary file 2 — Supplementary Material 2 [file 10886_2025_1624_MOESM2_ESM.docx]

**Fig. S1:** Number of *O. spinipes* females of chemotype 1 (orange) and chemotype 2 (violet) observed to be mated at specific ages (x-axis).


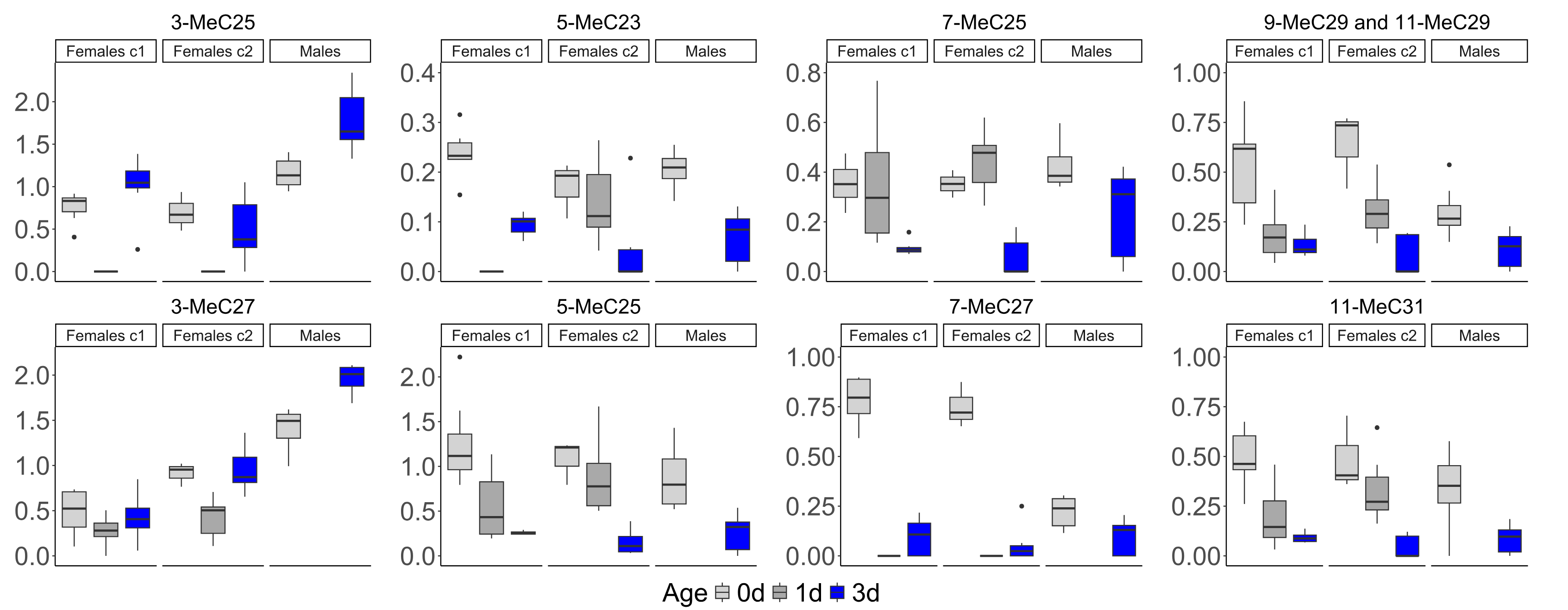


**Fig. S2**: Relative abundance of methyl-branched alkanes in *O. spinipes* males and females (c1 = chemotype 1; c2 = chemotype 2) on the day of eclosion (gray) and 3 days after eclosion (blue). The ordinate shows the relative abundance (in percent) of the different methyl-branched alkanes.


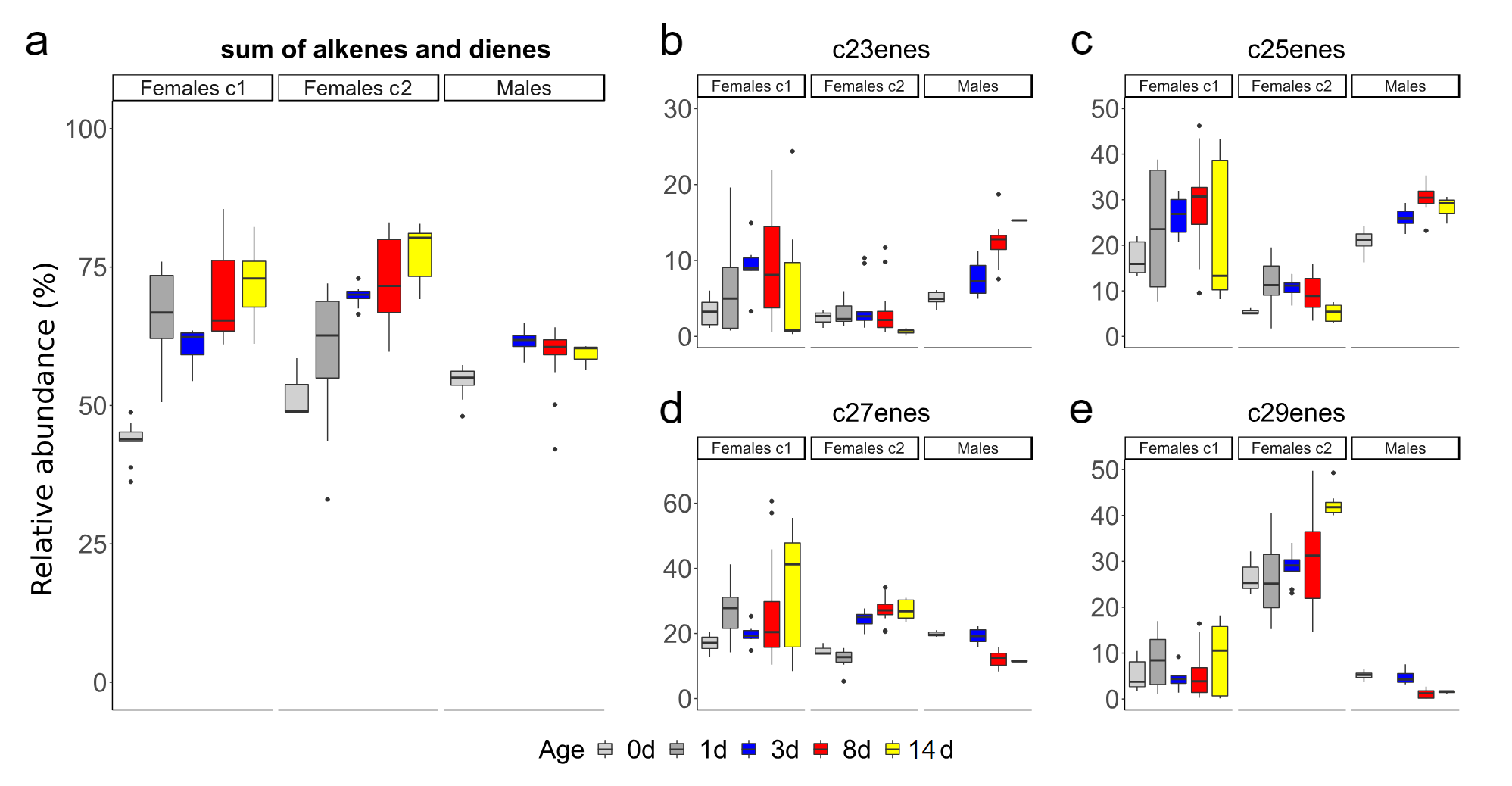


**Fig. S3**: Relative abundance of alkenes and alkadienes in *O. spinipes* females of chemotype 1 (Females c1); in *O. spinipes* females of chemotype 2 (Females c2) and in *O. spinipes* males (Males) at day 0 (grey), day 1 (dark grey), day 3 (bleu), day 8 (red), and day 14 (yellow). y axis is the relative abundance (in percent) of all alkenes and dienes (a), of all alkenes and dienes with a chain length of 23 carbons (b), of all alkenes and dienes with a chain length of 25 carbons (c), of all alkenes and dienes with a chain length of 27 carbons (d), of all alkenes and dienes with a chain length of 29 carbons (e).


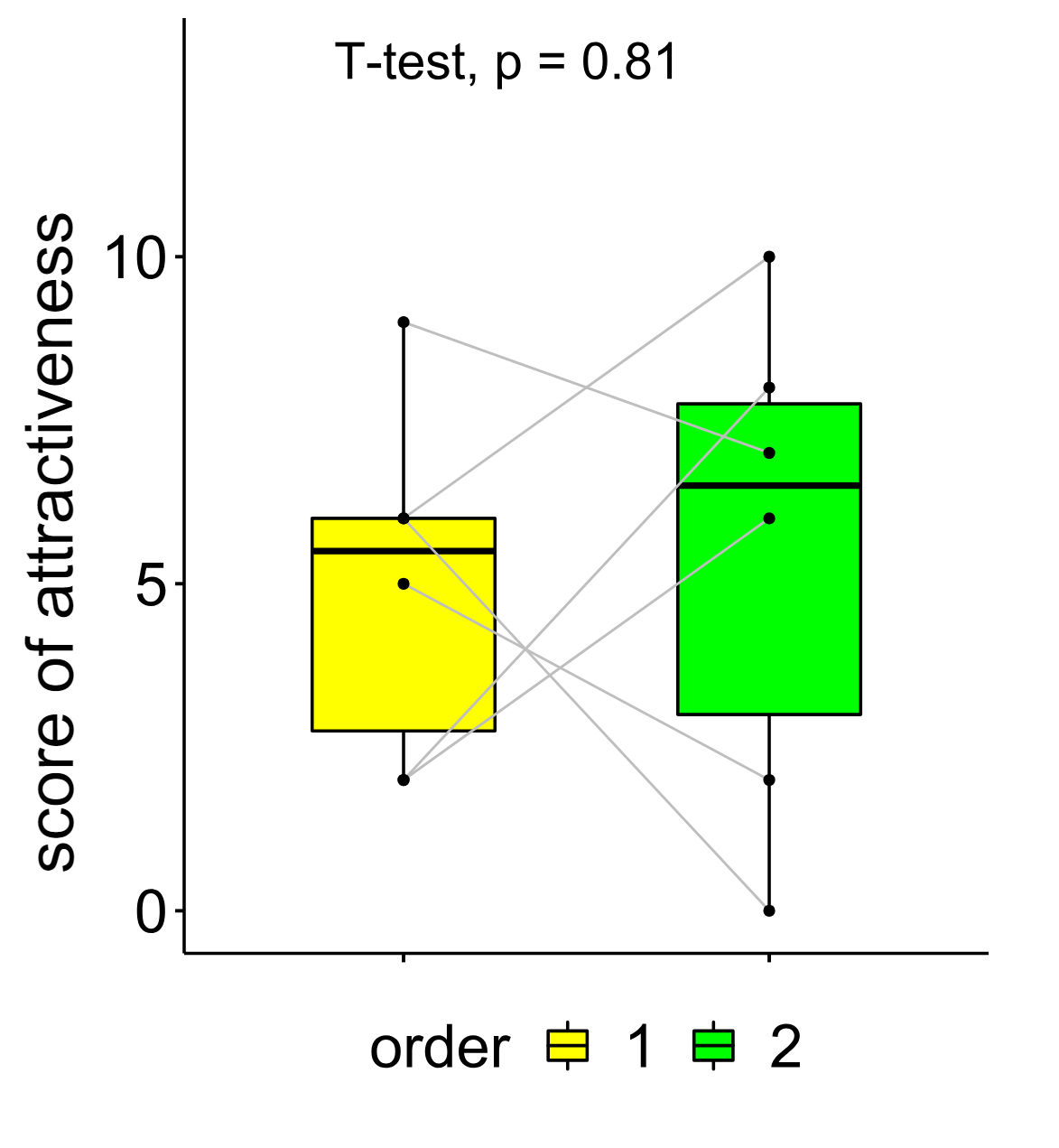


**Fig. S4:** Attractiveness scores of female dummies coated with cuticular hydrocarbons extracted from females presented to six males, as first dummy (yellow) and as second dummy (green). Gray lines indicate each pair of data. See Materials and methods for how attractiveness scores were calculated.
